# Heterogeneous climate change impacts on electricity demand in world cities circa mid-century

**DOI:** 10.1038/s41598-022-07922-w

**Published:** 2022-03-11

**Authors:** Yasmin Romitti, Ian Sue Wing

**Affiliations:** Boston University, Earth and Environment, Boston, 02215 USA

**Keywords:** Climate-change impacts, Energy supply and demand

## Abstract

Rising ambient temperatures due to climate change will increase urban populations’ exposures to extreme heat. During hot hours, a key protective adaptation is increased air conditioning and associated consumption of electricity for cooling. But during cold hours, milder temperatures have the offsetting effect of reducing consumption of electricity and other fuels for heating. We elucidate the net consequences of these opposing effects in 36 cities in different world regions. We couple reduced-form statistical models of cities’ hourly responses of electric load to temperature with temporally downscaled projections of temperatures simulated by 21 global climate models (GCMs), projecting the effects of warming on the demand for electricity circa 2050. Cities' responses, temperature exposures and impacts are heterogeneous, with changes in total annual consumption ranging from $-2.7$ to 5.7%, and peak power demand increasing by as much as 9.5% at the multi-GCM median. The largest increases are concentrated in more economically developed mid-latitude cities, with less developed urban areas in the tropics exhibiting relatively small changes. The results highlight the important role of the structure of electricity demand: large temperature increases in tropical cities are offset by their inelastic responses, which can be attributed to lower air-conditioning penetration.

## Introduction

Climate change is projected to increase the frequency and intensity of populations’ exposure to extreme high temperatures, particularly in urban areas^1^. Adaptation to such impacts will differ in cities across the world. Higher temperatures will adversely affect the health of urban populations^2^, and trigger adaptations that affect the demand for energy. The link between ambient temperature and mortality or morbidity is well understood^3,4^ and averting behavior via heating and cooling are a major driver of diurnal and seasonal variability in electricity demand^5,6^. Peak and total electricity demand are anticipated to increase worldwide as future warming increases demand for space cooling as an adaptation to more frequent and intense spells of high temperature^7^. Simultaneously, climate warming is projected to moderate cold-season low-temperature extremes, inducing reductions in the demand for electricity for space heating^8^. The relative magnitudes of these opposing effects will vary across locations and warming scenarios^9^. These stylized facts highlight the importance of quantifying (i) the impact on net energy demand of simultaneous increases in demand for cooling and decreases in demand for heating, (ii) the effectiveness of additional electricity use for cooling in moderating adverse heat-related health outcomes, and (iii) the environmental health, and welfare impacts associated with potential increases in greenhouse gases and other air pollutants emitted by the electric generation necessary to satisfy increased demand.

It has been estimated that climate change will stimulate a 25–58% increase global energy demand by mid-century^10^, but a key caveat is that this result is derived using coarse spatial (national) and temporal (annual) scale measures of both energy and temperature^11^. By contrast, populations’ exposures to extreme heat, and their decisions to consume energy as a means of shielding themselves from associated adverse health consequences, occur over finer time scales (hours to days) and geographic domains with a diversity of built environment contexts. Nationwide estimates are thus unlikely to capture the heterogeneity in demand response inherent across urban geographies resulting from distinct local structures of electricity demand and local climates. Global sub-national differences in urbanization and population growth further underpin the significance of evaluating the energy implications of local climate adaptation decisions that may be masked by national scale estimates^12^. Here we characterize heterogeneous local climate adaptation by leveraging fine spatial and temporal scale data to model the transient response of electricity to temperature. Our approach draws on substantial empirical literature on the effect of temperature on the demand for energy generally^11,13,14^, and electricity specifically^9,15–20^ to characterize the response of electric power consumption to temperature in 36 global cities on an hourly time step, and quantify the impact on high-frequency electricity demand of mid-century climate warming.

We make three contributions. First, data storage limitations have precluded archiving of global climate model (GCM) outputs at the hourly frequencies on which electricity load data have become available. Estimates of future heat exposures^12,21–25^ have largely relied on spatially downscaled GCM simulations of daily temperatures, but less attention has been paid to temporal downscaling to understand the transient impacts of warming temperatures. We address this gap by combining hourly temperatures from reanalysis data with daily mid-century temperature projections from the NASA Earth Exchange Global Daily Downscaled Climate Projections (NEX-GDDP) under two representative concentration scenarios (RCPs) of warming, 4.5 and 8.5, and applying a delta correction method^26,27^ to produce hourly current and mid-century climate temperature series. Second, we show that the hourly response of electricity demand to current and lagged temperatures leveraged in electricity market forecasting^28^ has important implications for adaptation, owing to varying thermal properties of the built environment^16^. Our statistical model incorporates a distributed lag structure similar to that employed in epidemiological investigations of temperature on mortality^29^. Our third contribution is bridging separate literatures that estimate climate impacts using differing techniques and scales. Econometric models of the nonlinear response of electric load to daily temperatures have been developed over broad spatial domains^18,30^ while recent estimates of high frequency cooling demand have combined high-resolution meteorological simulations with land use maps and simple transformations of load data for individual cities^31^. We improve upon these approaches by using hourly temperature observations to scale climate projections, and combining the results with empirically-derived impact response surfaces to estimate mid-century warming effects on electricity demand. Specifically, we employ hourly electricity data to econometrically estimate reduced-form statistical response surfaces of electricity demand to temperature in 36 global cities. We next temporally downscale GCM simulated future temperature projections by combining the diurnal cycle of hourly temperatures from ERA5^32^ with daily maximum and minimum temperatures from 21 GCMs in NEX-GDDP to construct hourly temperature series for current (2006-2020) and mid-century (2046–2060) climates that shed light on climatically-induced increases in urban extreme heat exposures and the demand for adaptation through cooling and electricity use. We combine these projections with our response surfaces to produce estimates of hourly impacts to electricity demand in 2050.

**Figure 1:**
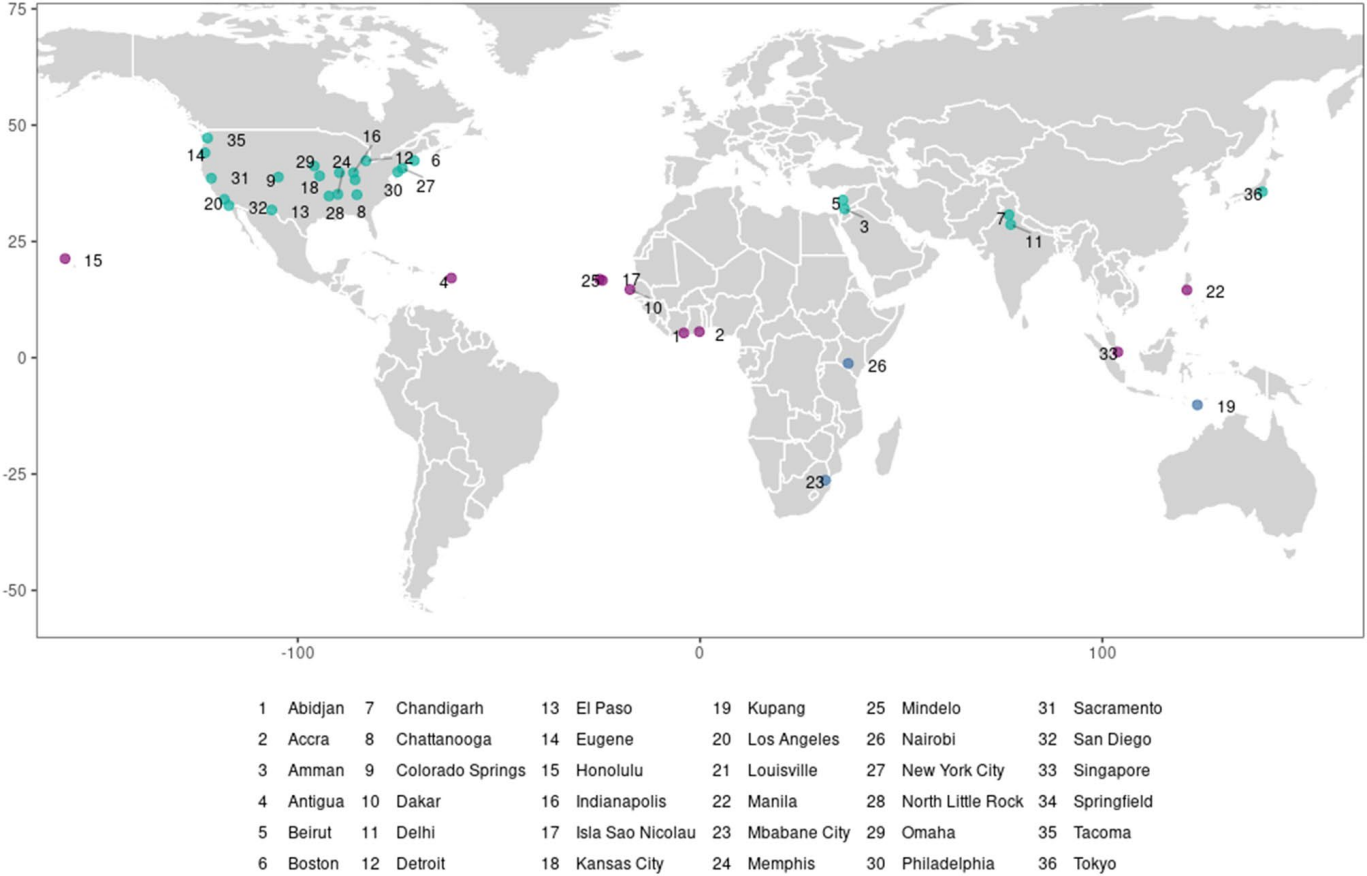
Geographical distribution of sample cities. Point color corresponds to category of demand response function: turquoise—“V”-shaped response, purple—increasing response, cyan—unresponsive.

The resulting heterogeneity in the transient response of electricity to temperature, and impact of future climate change on electricity demand among urban populations, differs with respect to both latitude and the structure of final demand for energy. Temperate cities largely exhibit “V”-shaped response functions: referring to the non-linear shape of the temperature-demand relationship that is well-documented in the empirical and engineering literatures^14,17,18,30,33^. Electricity demand for space cooling (heating) increases as temperatures rise above (fall below) the “set point” temperature, $T^*$, where weather-sensitive electric load, $L^*$, is minimized—typically between 10–30$^{\circ }$C. Tropical cities’ response functions range from increasing to unresponsive, as can be seen in Fig. 1, reflecting underlying differences in built environment characteristics and social and economic contexts across world cities. Furthermore, cities in mid-latitude temperate climates that currently experience cold winters and mild summers see reduced demand for a mix of fuels for heating but more electricity for cooling, with peak per-capita demand increases of up to 9.5% under future warming. Conversely, cities in the tropics that experience warm temperatures year-round vary in their responses, exhibiting both increased consumption of electricity for  cooling and overall reductions in demand, resulting in median per capita annual impacts ranging from $-2.7$ to 5.7% across GCMs. Projections of impacts under both future temperature shifts and demographic change represent $-4$ to 21% of mean annual historical demand across cities with the largest effects found in both the tropics and mid-latitudes. Annual impacts on electricity load further indicate that peak demand for cooling during warm periods may not be entirely offset by cool period reductions in demand. The diversity of responses in our results indicates a reversal of the north-south gradient of electricity demand impacts demonstrated in previous research^30^, which suggests the need for caution extrapolating impact responses derived for the mid-latitudes to locations in the tropics. While we are not able to observe additional attributes that may be responsible for intra-urban variation in energy demand (e.g., built environment heat transfer characteristics, prevalence of air conditioning), nor how developing cities may evolve in the future with urbanization, our results identify and characterize distinct patterns of temperature-responsive demand across cities, indicating these these areas are intriguing and merit further investigation.

## Results

### Heterogeneous demand response profiles

**Figure 2:**
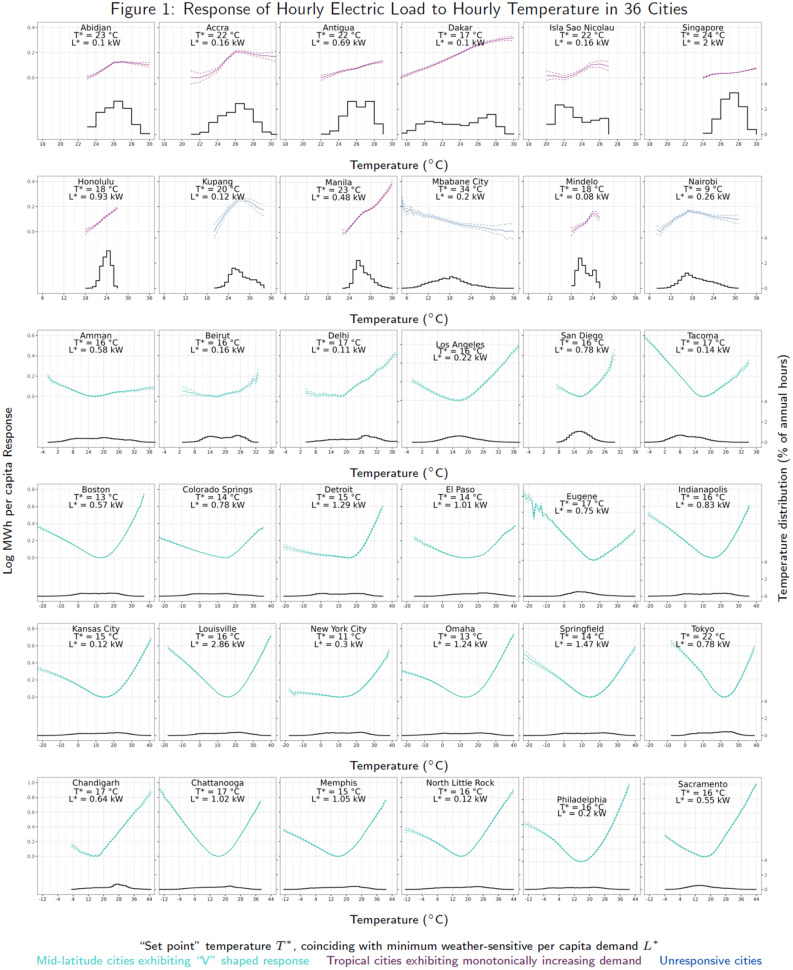
Response of hourly electric load to hourly temperature in 36 cities.

Figure S1 summarizes the responses of hourly electric load to current and lagged hourly temperatures as three-dimensional response surfaces (see Supplementary Information—SI for figure and details regarding basis for lag structure). Figure 2 illustrates the integrated response over contemporaneous and lagged temperatures, which falls into three broad categories: “V”-shaped, increasing, and unresponsive. “V”-shaped responses are characteristic of the mid-latitude temperate cities in our sample. For urban areas exhibiting this response, demand is generally largest for contemporaneous temperatures, is less responsive to temperatures experienced up to 4 hours prior, and, relative to this minimum point, is somewhat more responsive to temperatures further removed in time. These cities experience the widest range of seasonal temperatures ($-20$ to $+45$ $^{\circ }$C). By contrast, for most cities in the tropics, electricity demand increases with temperature—in some cases slightly non-monotonically (e.g., Abidjan, Accra, Mindelo). These climates are warm with small annual temperature ranges (17–30 $^{\circ }$C), which obviates the need for heating, with the result that $T^*$ coincides with the lower support of the temperature distribution. Here too, demand tends to be most responsive to contemporaneous temperatures, with temperatures at previous hours having impacts that are smaller in magnitude but vary considerably. Finally, three tropical cities (Mbabane City, Kupang, and Nairobi) exhibit patterns of electricity demand that are largely unresponsive to temperature, likely because of idiosyncratic geographic factors. (These areas experience a wide range of seasonal temperatures (5–35 $^{\circ }$C) and Mbabane and Nairobi are located at high altitudes with relatively mild climates, which may obviate the need for substantial cooling, whereas Kupang is located in the eastern portion of the Indonesian archipelago where electrification rates tend to be lowest^34^.)

### Climate change impacts, and drivers

Shifts in cities’ hourly temperature distributions due to climate change circa 2050 are constructed via the delta correction method by combining patterns of hourly temperature responses to diurnal extrema that we empirically estimate from reanalysis data with daily temperature extremes projected by 21 GCMs (see “Methods”). Figure S2 summarizes the resulting changes in mid-century cold- and hot-season temperature distributions for the RCP 8.5 warming scenario. (Distributions for RCP 4.5 are shown in Fig. S3.) Across GCMs, summer and winter hourly temperatures both increase for the majority of cities. The rightward movement in the upper support of the distribution of summer temperatures exceeds the shift in the median, indicating a lengthening of the tail of extreme high temperature exposures. Importantly, vigorous (RCP 8.5) warming’s induced shifts are larger for cities in the mid-latitudes (6–8$^{\circ }$C) compared to the tropics (5–6$^{\circ }$C), a result that contrasts with prior characterizations of climatic exposure such as the increase in the number of days with average temperatures above a pre-defined threshold^10^.

**Figure 3:**
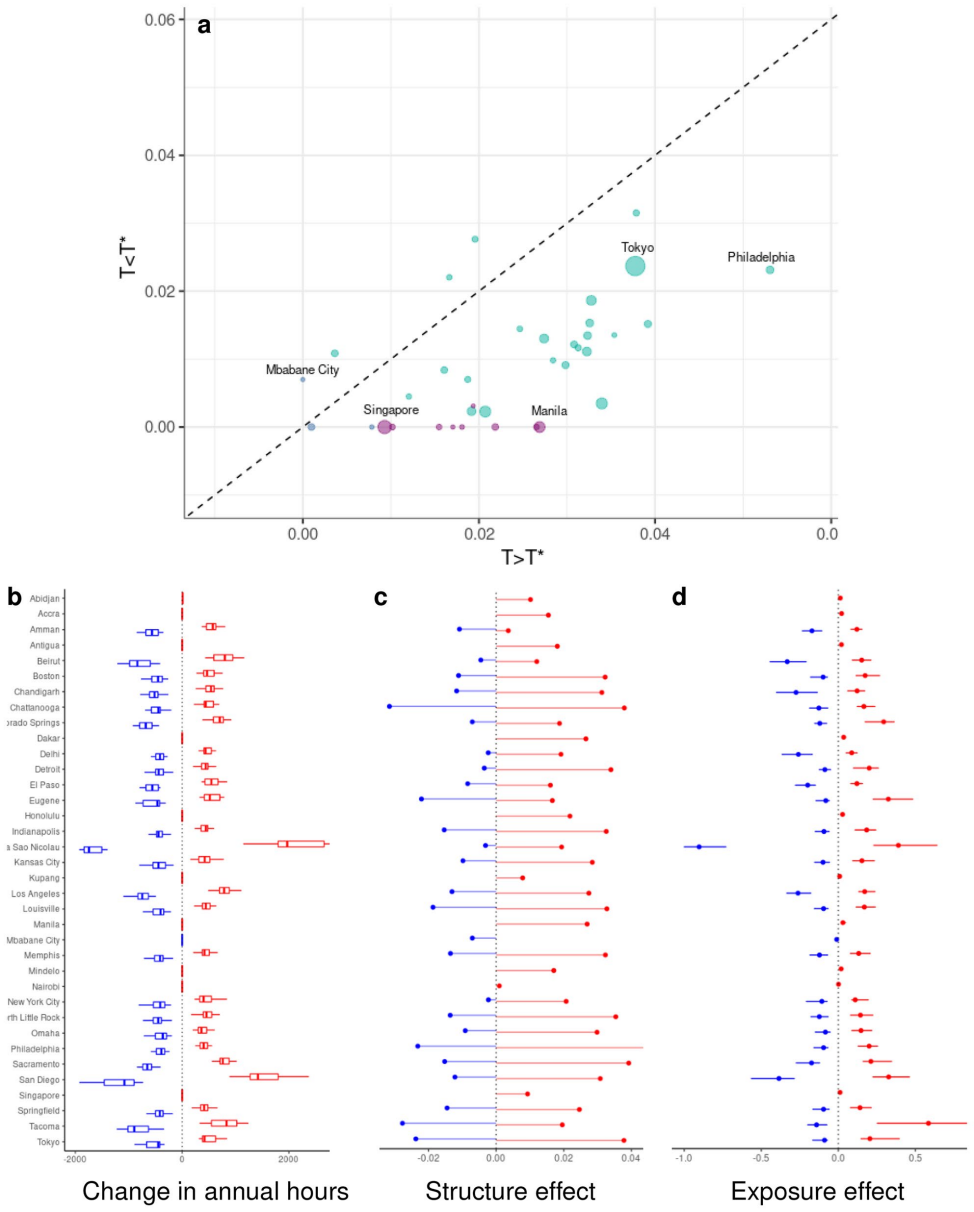
(**a**) Average fractional response of per capital urban electricity demand to temperatures above and below the set point temperature, $T^*$. Colors indicate type of demand response (turquoise—“V”-shaped, purple—increasing, cyan—unresponsive. (**b**) Change in hours over the year with temperatures above (red) and below (cyan) $T^*$. (**c**,**d**) Decomposition of the change in annual total electricity demand into the effects of demand response and shifts in the distribution of hourly temperatures.

To quantify the impact of mid-century warming on hourly electricity demand in each city, we force our estimated demand responses with projected temperature shifts and sum the resulting changes in hourly electricity demand per capita to project changes in electricity consumption on annual and sub-annual time-scales. Figure 3 decomposes shifts in total annual demand into the effect of climate change on the fractional change in total electricity demand for hours above and below $T^*$, and distinguishes the contribution of the structure of electricity demand (i.e., the magnitude of the average temperature semi-elasticity of demand) from the effects of the shift in temperature exposures (see “Methods”). In Panel a, populous mid-latitude cities such as Tokyo have historically large annual per capita load as well as a demand structure that is sensitive to both heating and cooling. Populous tropical developing-country cities such as Manila consume less electricity and exhibit temperature-inelastic heating electricity demand but highly elastic cooling electricity demand (see Table S1.) Panel b’s summary of the changes in annual hours above and below $T^*$ reveals a tendency toward increases in hot hours alongside commensurate or slightly smaller reductions in cold hours. (Exceptions tend to be tropical cities such as Abidjan, Dakar and Kupang where $T^*$ lies at the lower support of the temperature distribution in the current climate and remains there as the climate warms.) These influences drive the structure and exposure effects summarized in Panels c and d, for which the impacts of future temperature exposures dominate those of demand structure by an order of magnitude for many urban areas in our sample, primarily those in the mid-latitudes. In tropical cities where the vast majority of hours are hot, the structure and exposure effects are of similar magnitude—a phenomenon that is the key driver of net impact, to which we now turn.

**Figure 4:**
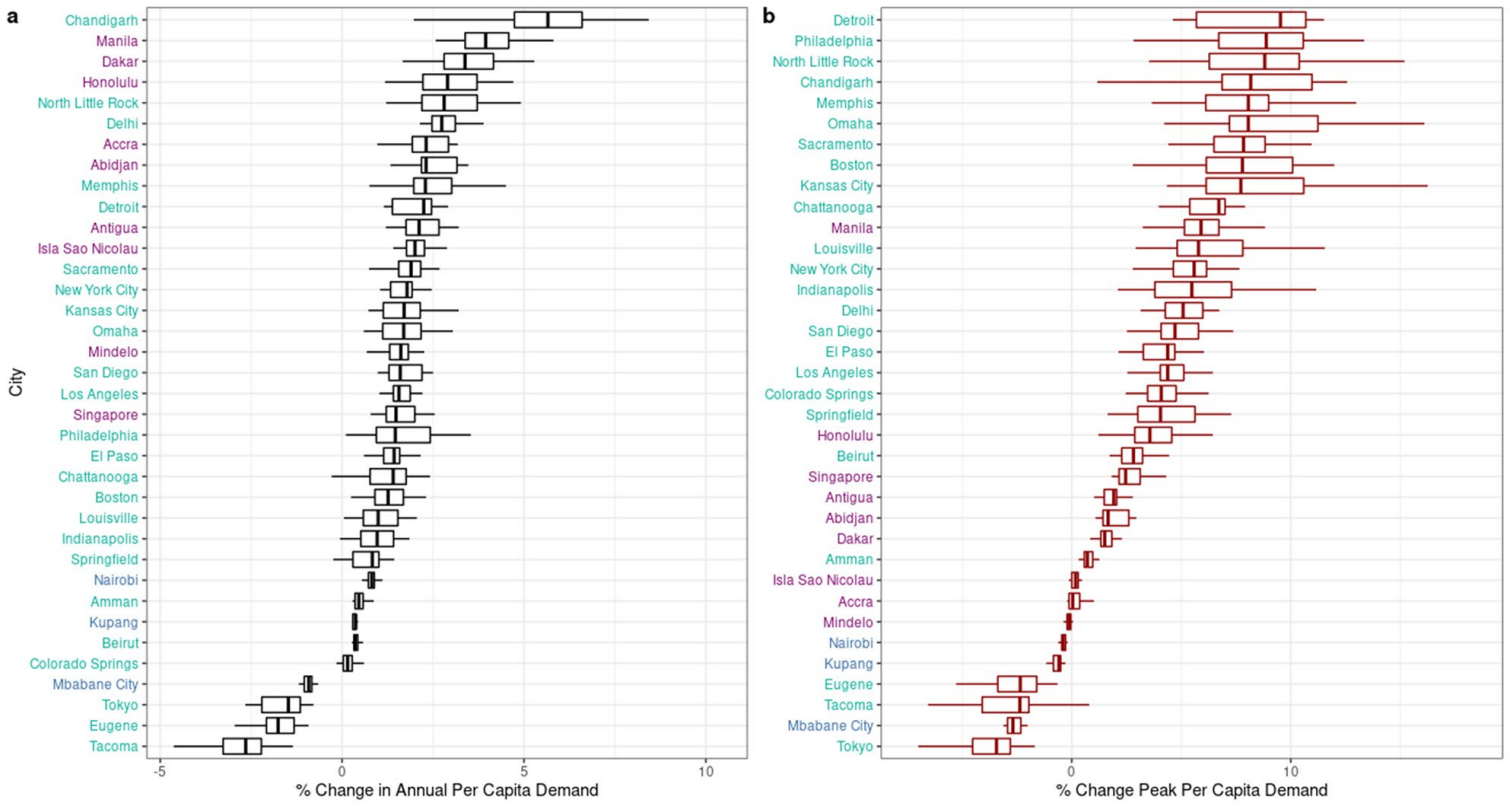
Percentage change in (**a**) total annual and (**b**) 95th percentile demand due to changes in hourly temperatures circa 2050, 21 GCM simulations of RCP 8.5 warming.

Figure 4 summarizes the consequent net impacts of climate change on the total and peak demand for electricity (Fig. S8 illustrates these impacts under RCP 4.5. Relative to the current climate, the change in annual demand per capita at the multi-model median ranges from $-2.7$ to $+5.7$% (Panel a), while the change in the multi-model median of the 95th percentile of hourly demand ranges from $-3.4$ to $+9.5$% (Panel b). In the majority of our sample of cities, the amplifying effect of the semi-elasticity of demand to hot temperatures ($T>T^*$) in conjunction with climatically-induced increases in hot hours outweighs the attenuating effect of the semi-elasticity of demand to cold temperatures ($T<T^*$) in conjunction with decreases in cold hours. The net of these effects is changes in demand of 0% up to +6% in 32 cities, and reductions of up to $-2.7$% in the remaining four cities. Within this latter group, mid-latitude cities (e.g., Tokyo, Eugene, and Tacoma) experience increases in hot hours that reduce electricity demand for heating by a larger amount than they increase demand for cooling, lowering net annual consumption. In Mbabane City, warming shifts hours from temperatures at which demand peaks to those where demand is actually smaller due to the decreasing monotonicity of its response.

These effects underlie changes in the 95th percentile of per capita demand (Panel b), which exceed $+5$% in nearly half of our sample, typically mid-latitude temperate cities in North America (e.g., Detroit: 9.5%, Philadelphia: 8.9%, North Little Rock: 8.8%), while among tropical cities, the largest increases are substantially smaller (e.g., Manila: $+5.9$%). By contrast, one fifth of the sample experiences *declines* in peak demand. Over-represented in this group are mid-latitude cities whose peak electricity demand occurs during the cold season, and the “unresponsive” tropical city of Mbabane. The surprising upshot is that the substantial increases in demand appear concentrated in mid-latitude cities, while demand changes experienced by urban areas in the tropics tend to be either increases or decreases of much smaller magnitude.

**Figure 5:**
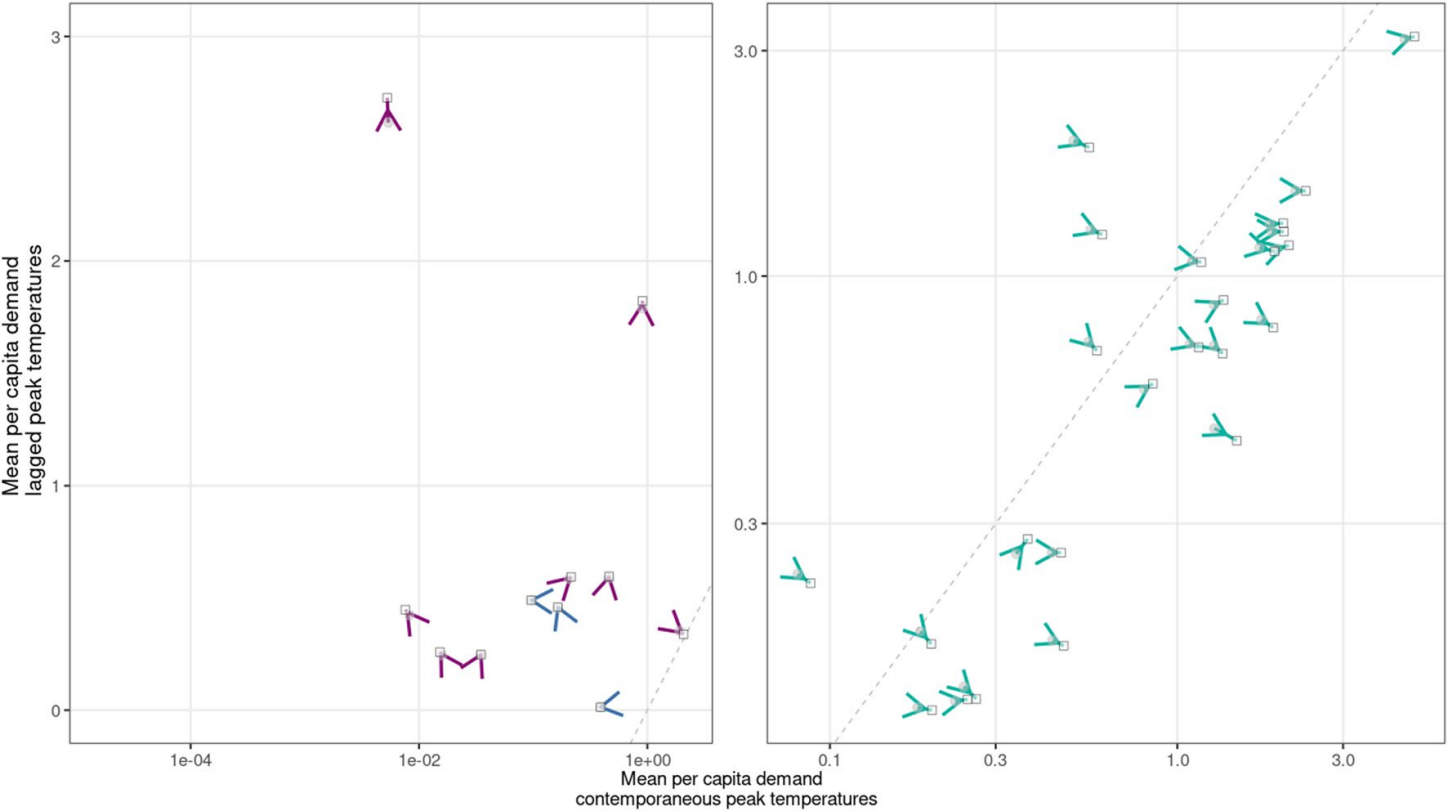
Change in demand components attributable to contemporaneous peak ($>95\text {th}$ percentile) temperatures (horizontal axis) and the cumulative effects of peak temperature exposures 1–6 h prior (vertical axis). Gray circles indicate mean MWh per capita demand during the historical period, hollow squares indicate median multi-model mean MWh per capita demand in 2050 under RCP 8.5, arrows indicate direction of change.

Heterogeneity in demand impacts also reflects the differing contributions of lagged versus current peak ($>95$th percentile) hourly temperatures in cities. Decomposing the cumulative effect of the prior 6 hours of peak temperature exposure on mean demand (lagged response) against the effect of current hourly peak temperatures (contemporaneous response) reveals that the latter is more influential in most cities in the mid-latitudes under both the current and 2050 climates, but generally not in the tropics, where instead the lagged response tends to dominate (Fig. 5, for RCP 4.5 see Fig. S9). For almost half of cities, climate change increases the effects of both the contemporaneous and the lagged response (upward pointing arrows). The remaining cities largely display simultaneous increases in the effect of the contemporaneous response and decreases in the effect of the lagged response (rightward pointing arrows), a divergence that arises from differences in the slopes of the demand response surface along the temporal dimension (Fig. S1).

**Figure 6:**
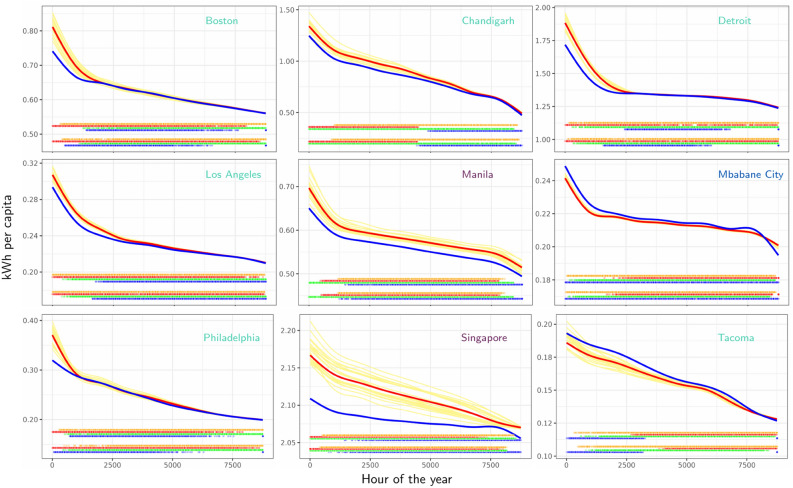
Current and projected 2050 load duration curves (LDCs) and seasonal distribution of load for a subset of cities. For each city, LDCs are shown for the current climate (blue), individual GCM simulations of RCP 8.5 warming (yellow), and the multi-GCM median (red). Colored panels illustrates each city’s seasonal distribution of hourly demand in the current climate (bottom) and RCP 8.5 warming (top), blue: winter, red: summer, green: spring, orange: fall.

Daily load profiles summarize the impact of climate change on hourly demand (Figs. S4 and S5). Mid-latitude cities experience up to a 20% (Philadelphia) increase in peak demand at certain hours. Load duration curves (LDCs) illustrate warming impacts to hourly demand and its seasonal dependence, a subset of which are shown in Fig. 6 (remaining city LDCs for RCP 8.5 are in Fig. S10, and for RCP 4.5 are in Fig. S11). Urban areas whose peak electricity consumption occurs during the hot season tend to experience amplification of peak demand (on the order of 0.02–0.42 kWh per capita), while those with cold- or shoulder-season peaks tend to experience peak demand attenuation. In locations such as Tacoma and Tokyo, the latter effect arises from the decline in winter peak demand with warming. In the tropics, cities such as Antigua, Honolulu and Manila experience amplification in load demand across all hours, while the remaining cities (which mostly belong to the “unresponsive” group) show either partial or almost complete reductions in demand over the course of the year (e.g., Mindelo and Mbabane City, respectively). For half of our sample of cities, concentrated in the mid-latitudes, cold-season hours shift lower in the rank-ordering of hourly demand. Only in a few of these locations is there a perceptible reverse shift of hot-season hours higher in the ordering (e.g., N. Little Rock). While in many cities, loads that correspond to hours in the shoulder seasons occur at all percentiles ranks, there is a slight tendency toward hours in spring (autumn) moving lower (higher) in the demand order, consistent with warming-induced electricity demand reductions for heating demand and increases for cooling.

Finally, we assess the implications of the foregoing impacts for urban areas’ absolute electricity consumption circa mid-century. Coupling our climatically-driven shifts in annual per capita load with 2050 population projections under two shared socioeconomic pathway (SSP) scenarios—SSP3 and SSP5 (see “Methods”)^35,36^, allows us to account for the additional influence of future population expansion (Table 1, RCP 4.5 Table S2). The RCP 8.5 multi-model median impact on annual load exceeds 500 GWh for a fifth of our sample of cities under both SSPs, with the largest load level increases in Manila, Singapore, Chandigarh, and Detroit—representing 7.2 (7.6)%, 2.5 (2.7)%, 20.8 (21.4)%, and 2.2 (3)% of annual demand for SSP3 (SSP5), respectively. The patterns of change in cities’ levels of annual demand are broadly consistent across both SSP scenarios, but larger in magnitude under SSP5. Effects are concentrated in mid-latitude temperate cities and electricity-intensive cities in the tropics (e.g., Manila, Singapore). At the 95$\text {th}$ percentile across GCMs, amplification of load over hot hours will require an additional 1000–3000 GWh in 8 cities.

**Table 1:** Change in total annual GWh across GCMs under RCP 8.5 at the median, 5th (low) and 95th (high) percentiles under SSP3 and SSP5. Median, 5th (low) and 95th (high) percentiles of change under both SSPs are also presented for future temperatures $\ge T^*$ and $< T^*$.

	SSP3								
	Annual			$T\ge T^*$			$T<T^*$		
	Median	Low	High	Median	Low	High	Median	Low	High
Abidjan	149.17	114.42	212.81	149.17	114.42	212.81			
Accra	285.67	208.74	385.25	285.67	208.74	385.25			
Amman	215.35	147.36	407.00	398.34	294.30	632.36	− 200.62	− 240.66	− 100.39
Antigua	18.04	13.56	26.35	18.04	13.56	26.35			
Beirut	7.26	5.37	10.32	7.13	5.55	10.24	− 0.08	− 0.25	0.15
Boston	196.56	71.57	332.30	357.80	185.20	516.34	− 153.84	− 274.21	− 51.45
Chandigarh	1248.60	669.16	1860.64	1241.10	668.98	1852.70	6.46	0.60	11.52
Chattanooga	78.27	− 1.45	122.43	160.82	116.24	193.83	− 85.27	− 145.77	− 20.71
Colorado Springs	7.19	− 4.41	28.61	41.41	25.40	62.59	− 33.98	− 47.54	− 19.71
Dakar	157.37	114.48	199.17	157.37	114.48	199.17			
Delhi	892.60	437.20	1344.47	865.22	417.48	1311.75	26.76	10.27	37.60
Detroit	1214.30	640.32	1526.11	1384.76	755.75	1645.97	− 133.07	− 260.83	− 67.80
El Paso	244.86	137.90	322.87	279.08	164.49	369.33	− 36.82	− 46.81	− 26.14
Eugene	− 43.19	− 71.62	− 24.92	4.87	1.96	8.01	− 47.91	− 79.00	− 26.93
Honolulu	262.45	127.59	380.82	262.45	127.59	380.82			
Indianapolis	121.04	32.31	205.56	291.84	168.20	421.35	− 162.77	− 296.45	− 70.78
Isla Sao Nicolau	0.63	0.44	0.91	0.58	0.35	0.88	0.06	0.01	0.09
Kansas City	38.03	17.06	66.60	55.97	33.35	96.88	− 17.23	− 30.11	− 10.14
Kupang	1.85	1.33	2.19	1.85	1.33	2.19			
Los Angeles	358.34	236.67	488.30	439.35	310.68	577.98	− 80.16	− 99.46	− 47.94
Louisville	359.80	71.39	643.23	838.94	572.27	1164.78	− 498.11	− 801.49	− 176.00
Manila	3712.61	2515.98	6016.88	3712.61	2515.98	6016.88			
Mbabane City	− 3.17	− 3.88	− 2.51				− 3.17	− 3.88	− 2.51
Memphis	317.27	132.76	542.01	415.88	241.83	680.49	− 101.67	− 156.00	− 31.01
Mindelo	1.40	0.96	1.95	1.40	0.96	1.95			
Nairobi	109.36	75.14	143.69	109.36	75.14	143.69			
New York City	937.79	554.71	1240.60	1030.00	585.30	1345.79	− 105.19	− 168.62	− 23.00
North Little Rock	17.10	8.17	27.45	23.15	13.73	35.85	− 5.23	− 7.82	− 2.31
Omaha	182.35	102.09	287.89	267.05	161.20	411.02	− 87.23	− 167.42	− 50.61
Philadelphia	164.18	12.76	336.56	461.03	261.40	555.25	− 265.09	− 424.85	− 45.73
Sacramento	163.16	100.00	230.45	205.34	137.64	316.86	− 56.70	− 79.81	− 28.30
San Diego	500.05	307.01	748.94	545.38	336.90	803.31	− 45.88	− 56.02	− 30.84
Singapore	2442.06	1617.88	3618.70	2442.06	1617.88	3618.70			
Springfield	27.73	− 1.41	47.16	58.66	36.27	102.88	− 36.65	− 68.23	− 18.29
Tacoma	− 42.26	− 71.35	− 25.56	0.27	− 0.44	1.77	− 42.10	− 71.62	− 25.73
Tokyo	− 2511.20	− 4297.67	− 1488.13	1402.82	761.27	2446.62	− 3739.96	− 6744.29	− 2625.65

	SSP5								
	Annual			$T\ge T^*$			$T<hT^*$		
	Median	Low	High	Median	Low	High	Median	Low	High
Abidjan	126.10	96.73	179.90	126.10	96.73	179.90			
Accra	267.09	195.17	360.20	267.09	195.17	360.20			
Amman	211.47	144.70	399.66	391.15	288.99	620.95	− 197.00	− 236.32	− 98.58
Antigua	18.04	13.56	26.35	18.04	13.56	26.35			
Beirut	6.44	4.76	9.16	6.33	4.93	9.09	− 0.07	− 0.22	0.13
Boston	267.79	97.51	452.73	487.46	252.31	703.46	− 209.59	− 373.59	− 70.09
Chandigarh	1280.43	686.22	1908.07	1272.74	686.03	1899.93	6.62	0.62	11.81
Chattanooga	110.06	− 2.03	172.17	226.15	163.47	272.58	− 119.92	− 205.00	− 29.12
Colorado Springs	10.08	− 6.17	40.08	58.00	35.58	87.68	− 47.60	− 66.60	− 27.61
Dakar	158.19	115.08	200.21	158.19	115.08	200.21			
Delhi	1031.99	505.47	1554.44	1000.34	482.68	1516.61	30.94	11.87	43.47
Detroit	1676.49	884.04	2106.98	1911.82	1043.41	2272.46	− 183.72	− 360.10	− 93.60
El Paso	255.46	143.87	336.85	291.16	171.61	385.32	− 38.42	− 48.84	− 27.27
Eugene	− 59.85	− 99.24	− 34.54	6.75	2.72	11.10	− 66.39	− 109.47	− 37.32
Honolulu	353.15	171.69	512.43	353.15	171.69	512.43			
Indianapolis	166.90	44.54	283.43	402.39	231.92	580.97	− 224.44	− 408.75	− 97.60
Isla Sao Nicolau	0.35	0.24	0.50	0.32	0.19	0.48	0.03	0.00	0.05
Kansas City	52.49	23.55	91.93	77.25	46.03	133.72	− 23.78	− 41.56	− 14.00
Kupang	2.03	1.46	2.41	2.03	1.46	2.41			
Los Angeles	490.70	324.10	668.67	601.63	425.44	791.48	− 109.77	− 136.20	− 65.66
Louisville	501.08	99.43	895.81	1168.37	796.98	1622.16	− 693.70	− 1116.21	− 245.12
Manila	3913.29	2651.98	6342.12	3913.29	2651.98	6342.12			
Mbabane City	− 3.12	− 3.82	− 2.47				− 3.12	− 3.82	− 2.47
Memphis	439.13	183.75	750.19	575.62	334.72	941.86	− 140.72	− 215.91	− 42.93
Mindelo	0.85	0.59	1.19	0.85	0.59	1.19			
Nairobi	126.43	86.87	166.11	126.43	86.87	166.11			
New York City	1270.88	751.74	1681.26	1395.85	793.19	1823.81	− 142.55	− 228.51	− 31.17
North Little Rock	23.73	11.34	38.08	32.12	19.06	49.74	− 7.25	− 10.85	− 3.21
Omaha	251.48	140.80	397.03	368.30	222.31	566.84	− 120.29	− 230.89	− 69.79
Philadelphia	223.92	17.40	459.03	628.78	356.51	757.29	− 361.55	− 579.45	− 62.37
Sacramento	224.58	137.64	317.21	282.64	189.45	436.14	− 78.04	− 109.86	− 38.96
San Diego	620.19	380.77	928.88	676.41	417.85	996.31	− 56.91	− 69.48	− 38.25
Singapore	2607.53	1727.51	3863.91	2607.53	1727.51	3863.91			
Springfield	39.56	− 2.01	67.27	83.67	51.74	146.75	− 52.28	− 97.33	− 26.09
Tacoma	− 57.86	− 97.70	− 35.00	0.37	− 0.60	2.43	− 57.64	− 98.07	− 35.23
Tokyo	− 3669.57	− 6280.11	− 2174.58	2049.91	1112.43	3575.20	− 5465.14	− 9855.31	− 3836.82

## Discussion

Across 36 cities, mid-century climate change will exert heterogeneous impacts on electric power consumption, driven by the interaction between shifts in cities’ distributions of hourly temperatures and the structure of their temperature-sensitive electricity demands. Increases in peak demand are concentrated in the mid-latitude, temperate, mostly North American cities in our sample, resulting in up to a 9.5% amplification at the 95th percentile of annual demand, while tropical cities’ peaks see comparatively modest increases (0–5.9%) and even declines (up to 0.6%). This pattern is corroborated by mid-century shifts in load duration curves, leading to increases in peak-hours of as much as 0.42 kW per person in North American cities—a substantial fraction of non-weather sensitive demand. The largest absolute increases in urban electricity demand are projected to occur in the tropics as well as the mid-latitudes, reflecting the importance of population expansion in addition to temperature-dependent per-person demand.

Our findings constitute an important qualification to the previously-discovered north-south polarization of electricity demand under climate warming^30^. Specifically, our results for the tropics suggest that this latitudinal gradient of impact severity is unlikely to extend all the way to the equator. The substantial temperature increases experienced by tropical cities induce shifts in demand that are much smaller than the impacts of only moderately larger temperature changes on mid-latitude urban areas, in contrast to prior empirically-based estimates of energy demand amplification under similar temperature change^10,18^. The difference is accounted for by prior studies’ coarser temporal resolution of temperature shifts and associated electricity demand (daily, annual), and is particularly an artifact of the thresholds used to bin daily temperatures in order to empirically establish their effects on temporally aggregated energy demand. Using our hourly temperatures to construct counts of hot ($\overline{T} \ge 27.5\,^\circ$C) days^10^, the apparent heat exposure gradient reverses to become substantially larger in the tropics (up to 149 additional hot days) in comparison to the mid-latitudes (a maximum of only 93 additional hot days) (Figs. S6 and S7). Although the magnitude of electricity demand responses to hot days for temperate regions has been found to exceed those for the tropics^11^, consistent with our results, their combination with binned temperatures leads to different patterns of impact across the globe^10^. Notwithstanding, our estimated increases in peak and total per capita energy demand for mid-latitude temperate cities mirror the findings of prior electricity-focused studies^18,37–39^.

We demonstrate that the character of the demand response to temperature, rather than solely the projected shift in the distribution of temperatures, is an important driver of heterogeneity in electricity demand amplification due to climate change. Despite the small size of our city sample, our results raise the caution that the “V”-shaped splines found by previous studies^11,40–43^ should not be considered universal, but instead tend to reflect the patterns of demand that prevail in richer, temperate, mid-latitude cities. The latter exhibit highly elastic responses to changes in temperature above and below $T^*$ that reflect built environment thermal properties different from those likely to prevail in the tropics. The corresponding 3D response surfaces (Fig. S1) consistently show high demand occurring at the hottest contemporaneous temperatures as well as the highest temperatures 6 hours prior, suggesting that buildings in these climates tend to hold heat very well. Tropical cities whose overall demand responses are linear and increasing exhibit a diversity of lag structures. This may be a feature of the quality of hourly demand data available in these cities (limited in temporal range and age of observations), though in addition, likely reflects not only climatic differences in built environment characteristics but also electrification and appliance availability that are correlated with economic development. Cities in countries further from the world technological frontier with less developed infrastructure tend to exhibit demand responses that are relatively temperature-inelastic at all lags, resulting in only moderate electricity consumption increases with warming, corroborating^42^. We also find moderate electricity demand impacts of future warming in most tropical cities, and show that they are attributable to relatively modest shifts in the temperature distribution on hourly scales. The tropics are home to a plethora of large cities whose income and population are projected to grow rapidly over the coming decades, but are under-represented in our sample. To understand how pervasive modest impacts might be, and establish the implications for future energy demand adaptation to climate change globally, will require concerted efforts to collect fine spatial and temporal scale data on energy demand in developing countries.

There are additional caveats to our findings. Perhaps the most important is our limited understanding of how the structure of electricity demand might change in the future. Evidence from India on the sensitivity of the shape of the demand response to increasing income^44^, reinforces the importance of this phenomenon. In view of the short periods over which our temperature-load relationships are estimated, we are unable to draw inferences about the manner in which these responses are likely to shift over time with economic development and technological progress^11^. Our projections thus indicate how temperature changes might influence electricity demand, holding the characteristics of urban areas constant as they are today. An alternative approach could be to utilize the response of mid-latitude cities in developed nations as models for the future, on the assumption that they reflect the manner in which tropical cities might evolve on the time frames over which the climate is anticipated to change^30^. If tropical cities with relatively inelastic demand responses are endowed with the response function of the tropical city with the most weather-sensitive demand in our sample (Manila), future warming induces larger percentage (up to $+5.3$%) and absolute (up to 798 GW under SSP5) changes in peak load (Fig. S12 and Table S3). Nevertheless, whether tropical developing urban areas at mid-century will behave similarly to their mid-latitude developed counterparts today is highly uncertain. Temperate cities may provide an indication for how the set point of the temperature-responsive components of demand may shift, however, this assumption does not indicate how the overall shape of cities’ demand responses might shift with economic and technological development. To address this gap, one potential avenue for future research is analyzing how the shape of the temperature-load relationship shifts with proxies for structural and technological change in energy systems that can be linked to scenarios of socioeconomic futures^45^. Given the dependence of demand in the least developed regions on the diffusion of electrification^46^, the challenge will be to develop sufficiently long load time series from which to infer how demand responses to temperature might be influenced by development, and use the results as archetypes that can be applied to electrifying locations.

A related shortcoming, shared with prior empirical work^10,11,18,30^, is our model’s implicit focus on adaptation at the intensive margin—the temperature responsiveness of energy demand conditional on the stock of electricity-using durable goods, without explicit consideration of the extensive margin—temperature-induced technology adoption, particularly air conditioning (AC) capital^47^. The latter has been quantified by exploiting variation in stocks of energy-using durables across locations with different climates and incomes, in conjunction with spatially and temporally varying data on electricity demand and weather^48^. This approach has leveraged observations of multiple locations within a country, but given the large differences in the levels of economic development among our cities and in particular the paucity of data on appliance holdings, its relevance to the current analysis remains open to question. A potential way forward is characterizing the joint dependence of AC penetration on temperature, income and other indicators of development leveraging census data for temperate and tropical countries. This is an active area of our research in progress.

Despite these limitations, our contribution is to highlight new implications for climate change impacts and adaptation of the fine temporal and spatial scale structure of electricity demand. Our results suggest that prior demand responses estimated for developed, temperate areas are unlikely to reflect the temperature dependence of electricity consumption in developing, tropical areas. Naive extrapolation of patterns of energy demand adaptation to warming observed in the mid-latitudes could thus potentially over-estimate the magnitude of climate change impacts, both in the tropics and globally. Even so, we urge caution in drawing broad conclusions from the current small sample of cities. As primary data collection makes available additional hourly load series in diverse areas that have not been previously observed, our bottom-up methodology serves as a template that facilitates quantification of the net effects of temperature on electricity demand associated with heterogeneous climatically-induced rightward shifts in local temperature distributions. Over time, expanding the slate of observations to a more diverse array of urban areas will enhance understanding of the extent to which top-down estimates of the energy demand impacts of climate change might be constrained by fine-scale heterogeneity in climatic and socioeconomic conditions.

## Methods

We empirically project the changes in electricity demand in urban areas as a consequence of mid-century climate change by first using hourly observations of temperature and electricity load to model urban electricity demand response functions. Separately, we use daily temperature series simulated by 21 GCMs and observed historical hourly temperature series in conjunction with a temporal downscaling algorithm to calculate the shifts in extreme low and high temperature exposures that will be experienced by these 36 global cities to 2050. The projected shifts in temperature distribution are combined with results from the demand response functions to construct projections of climate-change driven shifts in hourly electricity demand.

### Data

For use in our empirical model, we first combine data of hourly electricity demand with meteorological data and population estimates across 36 global cities. Hourly electricity consumption profiles for the majority of cities in this data sample were taken from Cohen et al.^49^ converted to Universal Coordinated Time (UTC) and matched with corresponding temperature values. The dataset provides hourly electric load series in megawatts (MW) for various years spanning the period 2003-2018 across different urban areas in North America, Africa, and (primarily southern) Asia (Table S4). While its geographic coverage is limited, the dataset's fine spatial (city) and temporal (hourly) resolution distinguishes it from similar datasets of hourly electric load that are only available for broad spatial domains (e.g., U.S. load balancing areas or European countries).

As described in^42^, hourly electricity demand was collected from electric utilities, system operators and regulatory bodies serving sample cities for the most recent years for which data were available at the time, as such these data may not reflect the most current state of these urban energy systems. For India's National Capital Territory of Delhi, load was disaggregated into five “cities”, each served by a distinct and non-overlapping electricity distribution company (details in^42^, Supplementary Information). We re-aggregate these series to provide summed hourly profiles of electricity consumption for the entire Delhi urban area. Further information describing load data and quality are available in the SI.

Meterological data are obtained from the European Centre for Medium-Range Weather Forecasts’ (ECMWF) ERA5 dataset^32^. Centroids of cities’ central business districts or financial districts (where available) were taken to be representative of their encompassing urban area, and used to query ERA5 raster datasets. We extracted hourly series for 2 m air temperature, dew point temperature, wind speed, and downward shortwave radiation for years matching our electricity load data. To construct hourly measurements from GCM daily minimum and maximum temperature projections, hourly air temperatures were extracted for all cities for the 15 year period 2004–2018.

We utilize the NASA Earth Exchange Global Daily Downscaled Projections (NEX-GDDP) to calculate the effects of mid-century temperature projections relative to those in the historical period^50^. NEX-GDDP contain bias-corrected and spatially downscaled daily maximum and minimum temperatures on a 0.25$^{\circ }$ grid over the 2006-2100 period for RCP 4.5 and RCP 8.5 scenarios derived from 21 GCMs participating in the global Climate Model Intercomparison Project phase 5 (CMIP5)^51^. Detailed information on NASA NEX-GDDP is available at https://cds.nccs.nasa.gov/nex-gddp/ and described in both https://esgf.nccs.nasa.gov/esgdoc/NEX-GDDP_Tech_Note_v0.pdf and^50^.

Population data were collected from the U.S. Bureau of Economic Analysis (BEA) Personal Income, Population, Per Capita Personal Income (CAINC1) tables for all U.S. cities in our sample by metropolitan statistical area (MSA)^52^. For cities with populations >300,000, population estimates were obtained from the United Nations World Urbanization Prospects 2018 revision^53^. Estimates for Antigua, Isla Sao Nicolau, Kupang, Mbabane City, and Mindelo were gathered from both national and international sources; described in Table S8. Spatially explicit global population projections consistent with the Shared Socioeconomic Pathways (SSPs) are available from^54^. These projections cover the period 2010–2100 in ten year time intervals on a 0.125$^\circ$ grid. We extract mid-century population projections for SSP3 (high challenges to mitigation and adaptation) and SSP5 (high challenges to mitigation, low challenges to adaptation) to scale our projections of average hourly per capita impact to electricity demand. SSP3 describes a demographic trajectory characterized by high population growth rates in the high fertility countries and declining population in low fertility countries with slow rates of urbanization, while SSP5 describes a scenario of accelerated demographic transition with low population growth in high fertility countries, high levels of growth in low fertility countries, and rapid urbanization^36,54^.

### Empirical approach

We estimate the nonlinear response of cities’ electricity load to temperature using a generalized additive model (GAM)^55^. Letting *h* index hours of the year, electric load ($L_h$) for each of 36 cities is modeled as a crossbasis function of matched hourly current and lagged local temperatures ($T_h$) from ERA5 using the regression

1 $$\begin{aligned} \ln L_h = \Psi [T_h, T_{h-1}, \ldots , T_{h-\ell }; \varvec{\theta }] + \text {controls} + u_h \end{aligned}$$

where $\Psi [\cdot ]$ denotes a smooth tensor product parameterized by a vector of estimated coefficients, $\varvec{\theta }$, and $u_h$ is a random disturbance term. We set the maximum lag length, $\ell$, to six hours to capture the effects of unobserved heat fluxes in the built environment and the thermal properties of buildings and structures^56^. (See SI: Methods and Data for a detailed explanation of the theoretical basis for lag structures in building energy use.) Statistical controls include meteorological predictors of built environment heat transport and thermal performance (ERA5 hourly downward solar radiation, dew point temperature, and wind speed), as well as parsimonious proxies for unobserved non-meteorological determinants of demand (including a smooth function for time and a categorical year dummy that captures the energy consumption impacts of households’ and firms’ routines on various time scales). The fitted response, $\widehat{\Psi }=\Psi [\cdot ;\widehat{\varvec{\theta }}]$, exhibits its lowest point at the “set point” temperature, $T^*$, at which weather-sensitive electricity consumption is minimized, $L^*$. Following from results in^30,42^, we offset $\widehat{\Psi }$ by the amount $\min (\ln L(T))$ for $10\,^\circ \text {C} \le T \le 30\,^\circ$C. This allows us to interpret $\widehat{\Psi }$ as the fractional increase in electricity demand above $L^*$ per $^{\circ }$C ambient temperature above or below $T^*$. For most cities, $\Psi$ is approximately linear on either side of $T^*$ (Fig. 2)^18^, which allows us to represent the average slopes of the responses as the parameters $\beta _C$ and $\beta _H$ for $T < T^*$ and/or $T > T^*$, respectively.

### Hourly temperature projections

Projections of current and future electric load utilize daily maximum and minimum temperatures simulated by 21 GCMs from the NASA NEX-GDDP for RCPs 4.5 and 8.5 scenarios. We use ERA5 observed temperatures to estimate the hourly pattern corresponding to diurnal extrema, $T_d^{Max}$ and $T_d^{Min}$, where *d* indexes the day of year. We assume a stable relationship between hourly temperatures and daily extremes, representing $T_h$ as the convex combination of the maximum and minimum:

2 $$\begin{aligned} T_{h,d} =\omega _h T_d^{Max} + (1 - \omega _h) T_d^{Min} \end{aligned}$$

in which the hour-specific weight, $\omega _h$, is to be determined. Our empirical implementation of this approach is the linear regression

3 $$\begin{aligned} T_{h,d}-T_d^{Min}=\omega _h (T_d^{Max}-T_d^{Min})+v_d \end{aligned}$$

where *v* is a Gaussian error term. Equation (3) is estimated separately for each hour of the day for individual months, to fully account for interannual variation in climate. The fitted values of the weights, $\widehat{\omega }_h$, are then used to temporally downscale NEX-GDDP daily temperature extremes ($T_d^{Min,k}$ and $T_d^{Max,k}$, where $k\in \lbrace \text {Current},\text {Future} \rbrace$ is an index of current (2006–2020) and future (2045–2059) periods). The result is the synthetic hourly temperature series:

4 $$\begin{aligned} T_{h,d}^k=\widehat{\omega }_h T_d^{Max,k}+(1-\widehat{\omega }_h)T_d^{Min,k} \end{aligned}$$

We then apply the delta correction technique^57^: taking the difference in the period-means of synthetic temperature at each hour of the year $(\widetilde{T}_{h,d}^k)$, and adding the result to the mean of the temperature at each hour of the year in the current period from ERA5 $(\overline{T}_h)$,yields our estimate of average hourly temperature under warming:

5 $$\begin{aligned} \widetilde{T} _{h,d}=(\overline{T} _{h,d}^{Future}-\overline{T} _{h,d}^{Current})+\overline{T} _h \end{aligned}$$

### Climate change impacts

Our primary metric of the impact of climate change on hourly electricity demand is

6 $$\begin{aligned} I_{h,d}=L^*\times (\exp {\widehat{\Psi }[\widetilde{T}_{h,d}]}-\exp {[\widehat{\Psi }[\overline{T}_{h,d}]}) \end{aligned}$$

the results of which are summarized in Figs. 4 and 5. We sum the average hourly per capita impact over all hours and scale the result by future population in the SSP3 and SSP5 scenarios to project impacts on annual total electricity demand (Table 1).

Transforming our hourly temperature series into the distribution, *H*(*T*), which denotes the count of hours over the year in each $1\,^\circ$C bin of temperature, the total annual demand for electricity over cold and hot hours can be expressed as:

$$\begin{aligned} Q|_{T < T^*} \approx \sum _T L^* \exp \lbrace \beta _C (T^* - T) H(T) \rbrace \quad \text {and} \quad Q|_{T > T^*} \approx \sum _T L^* \exp \lbrace \beta _H (T - T^*) H(T) \rbrace . \end{aligned}$$

Our second impact metric decomposes the effect of climate change on the fractional change in total electricity demand for hours above and below $T^*$:

7 $$\begin{aligned} \frac{1}{Q|_{T< T^*}} \frac{\partial Q|_{T < T^*}}{\partial T}&\approx \beta _C + \sum _T \left[ \frac{L^* \exp \lbrace \beta _C (T^* - T) \rbrace }{\sum _T L^* \exp \lbrace \beta _C (T^* - T) H(T) \rbrace } \right] \cdot \left[ \frac{1}{H(T)} \frac{\partial H(T)}{\partial T} \right] \end{aligned}$$

8 $$\begin{aligned} \frac{1}{Q|_{T> T^*}} \frac{\partial Q|_{T > T^*}}{\partial T}&\approx \beta _H + \sum _T \left[ \frac{L^* \exp \lbrace \beta _H (T - T^*) \rbrace }{\sum _T L^* \exp \lbrace \beta _H (T - T^*) H(T) \rbrace } \right] \cdot \left[ \frac{1}{H(T)} \frac{\partial H(T)}{\partial T} \right] \end{aligned}$$

The first right-hand side term captures the effect of the structure of electricity demand—the percentage increase in demand from an additional hour, dependent on the magnitude of the slope of the response. The second term captures the effect of the change in exposure due to the shift in the temperature distribution—the demand-weighted average percentage change in hours. These two effects are summarized in Fig. 3, Panels c and d.

## Supplementary Information

Supplementary Information.

## Data Availability

The data generated during our analyses and supporting the findings of this paper are available from the corresponding author upon reasonable request. The input data used in this analysis are publicly available at the following: urban energy demand data: https://github.com/Ecohen4/Energy, other sources listed in Table S7. ECMWF ERA5 reanalysis data: https://www.ecmwf.int/en/forecasts/datasets/reanalysis-datasets/era5. NASA-NEX climate data: https://cds.nccs.nasa.gov/nex-gddp/. Population data: listed in Table S8. Spatial population projections for the SSPs: https://www.cgd.ucar.edu/iam/modeling/spatial-population-scenarios.html.
