# Author Correction: Heterogeneous climate change impacts on electricity demand in world cities circa mid-century

**DOI:** 10.1038/s41598-022-09077-0

**Published:** 2022-03-25

**Authors:** Yasmin Romitti, Ian Sue Wing

**Affiliations:** Boston University, Earth and Environment, Boston, 02215 USA

Correction to: *Scientific Reports* 10.1038/s41598-022-07922-w, published online 11 March 2022

The original version of this Article contained an error in the Acknowledgments section.

“This work was supported by the Frederick S. Pardee Center for the Study of the Longer-Range Future and National Science Foundation Research Traineeship (NRT) Grant No. DGE 1735087 (Y.R.), and by the U.S. Department of Energy, Office of Science, Biological and Environmental Research Program, Earth and Environmental Systems Modeling, MultiSector Dynamics, Contract No. DE-SC0016162 (I.S.W. and Y.R.).”

now reads:

“This work was supported by the Frederick S. Pardee Center for the Study of the Longer-Range Future, the Boston University Initiative on Cities, and National Science Foundation Research Traineeship (NRT) Grant No. DGE 1735087 (Y.R.), and by the U.S. Department of Energy, Office of Science, Biological and Environmental Research Program, Earth and Environmental Systems Modeling, MultiSector Dynamics, Contract No. DE-SC0016162 (I.S.W. and Y.R.).”

The original Article has been corrected.

