# Supplementary material for: Heterogeneous climate change impacts on electricity demand in world cities circa mid-century
Source: Sci Rep. 2022 Mar 11;12:4280. doi: 10.1038/s41598-022-07922-w (PMC8917203; doi:10.1038/s41598-022-07922-w)
Supplement: Supplementary file 1 — Supplementary Information. [file 41598_2022_7922_MOESM1_ESM.docx]

Supplementary Information

**Midcentury Temperatures Lead to Heterogeneous Amplification of Urban Electricity Demand Worldwide**

Y. Romitti and I. Sue Wing

**Table S1**

City demand response function slopes and mean historical annual load

| **City** | **Load (MW)** | **T>T*** | **T<T*** |
| --- | --- | --- | --- |
| Abidjan | 3648083 | 0.01 | 0 |
| Accra | 3060537 | 0.015 | 0 |
| Amman | 8559818 | 0.004 | -0.011 |
| Antigua | 690691 | 0.018 | 0 |
| Beirut | 2431659 | 0.012 | -0.004 |
| Boston | 25280227 | 0.032 | -0.011 |
| Chandigarh | 5996238 | 0.031 | -0.012 |
| Chattanooga | 5849679 | 0.038 | -0.032 |
| Colorado Springs | 4701770 | 0.019 | -0.007 |
| Dakar | 2977677 | 0.027 | 0 |
| Delhi | 24355322 | 0.019 | -0.002 |
| Detroit | 55832185 | 0.034 | -0.003 |
| El Paso | 7961533 | 0.016 | -0.008 |
| Eugene | 2585002 | 0.017 | -0.022 |
| Honolulu | 7969114 | 0.022 | 0 |
| Indianapolis | 15960740 | 0.033 | -0.015 |
| Isla Sao Nicolau | 5525 | 0.019 | -0.003 |
| Kansas City | 2505233 | 0.028 | -0.01 |
| Kupang | 337048 | 0.008 | 0 |
| Los Angeles | 27030698 | 0.027 | -0.013 |
| Louisville | 36591983 | 0.033 | -0.019 |
| Manila | 51609574 | 0.027 | 0 |
| Mbabane City | 79579 | 0 | -0.007 |
| Memphis | 15034468 | 0.032 | -0.013 |
| Mindelo | 62972 | 0.017 | 0 |
| Nairobi | 8274898 | 0.001 | 0 |
| New York City | 54064286 | 0.021 | -0.002 |
| North Little Rock | 1013579 | 0.035 | -0.014 |
| Omaha | 10907287 | 0.03 | -0.009 |
| Philadelphia | 15028181 | 0.053 | -0.023 |
| Sacramento | 11404983 | 0.039 | -0.015 |
| San Diego | 9319343 | 0.031 | -0.012 |
| Singapore | 98524496 | 0.009 | 0 |
| Springfield | 3276097 | 0.025 | -0.014 |
| Tacoma | 4958091 | 0.02 | -0.028 |
| Tokyo | 2.66E+08 | 0.038 | -0.024 |


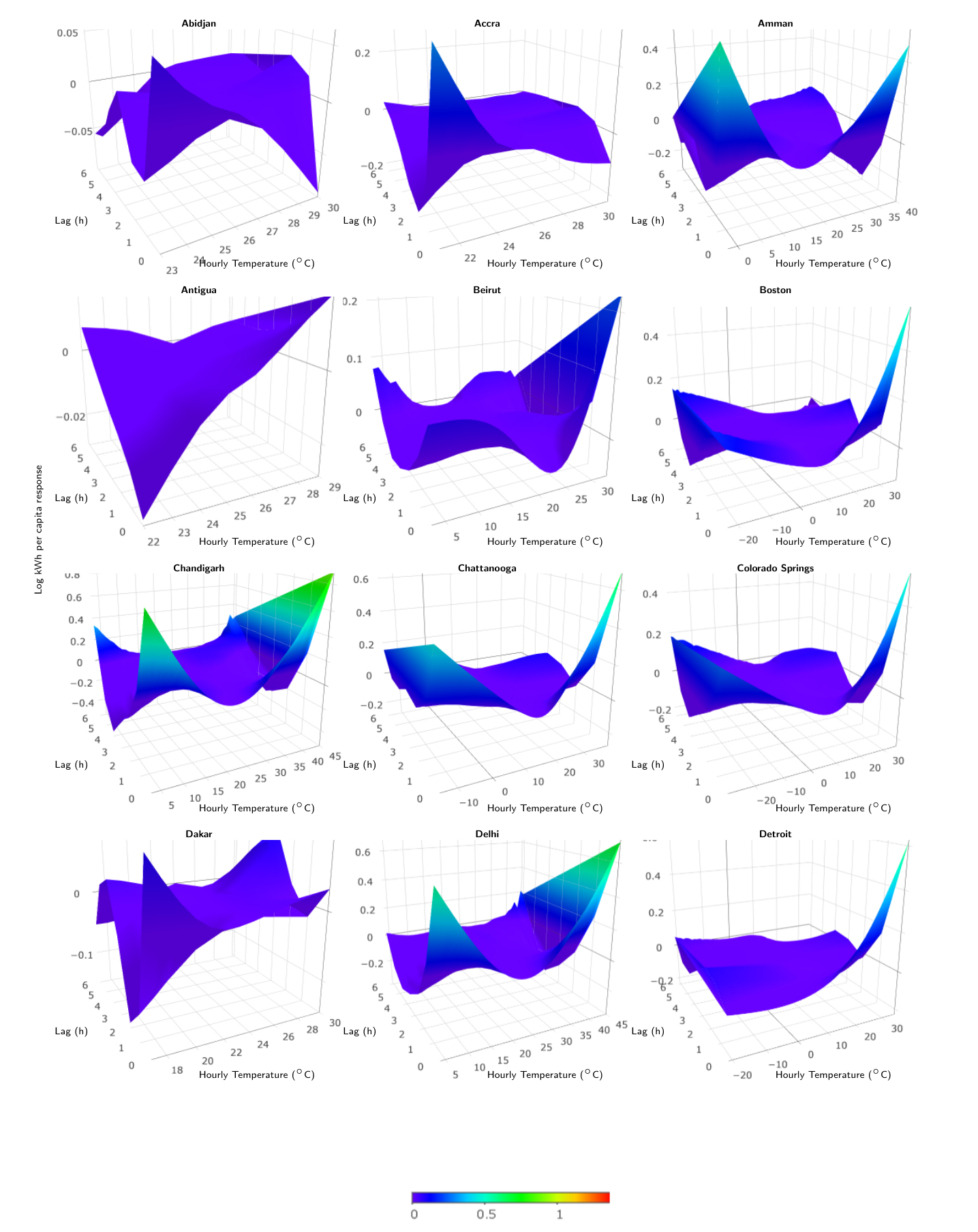
**Figure S1** 3D response surfaces of per capita load to current and lagged hourly temperatures

**Figure S1 (continued)**


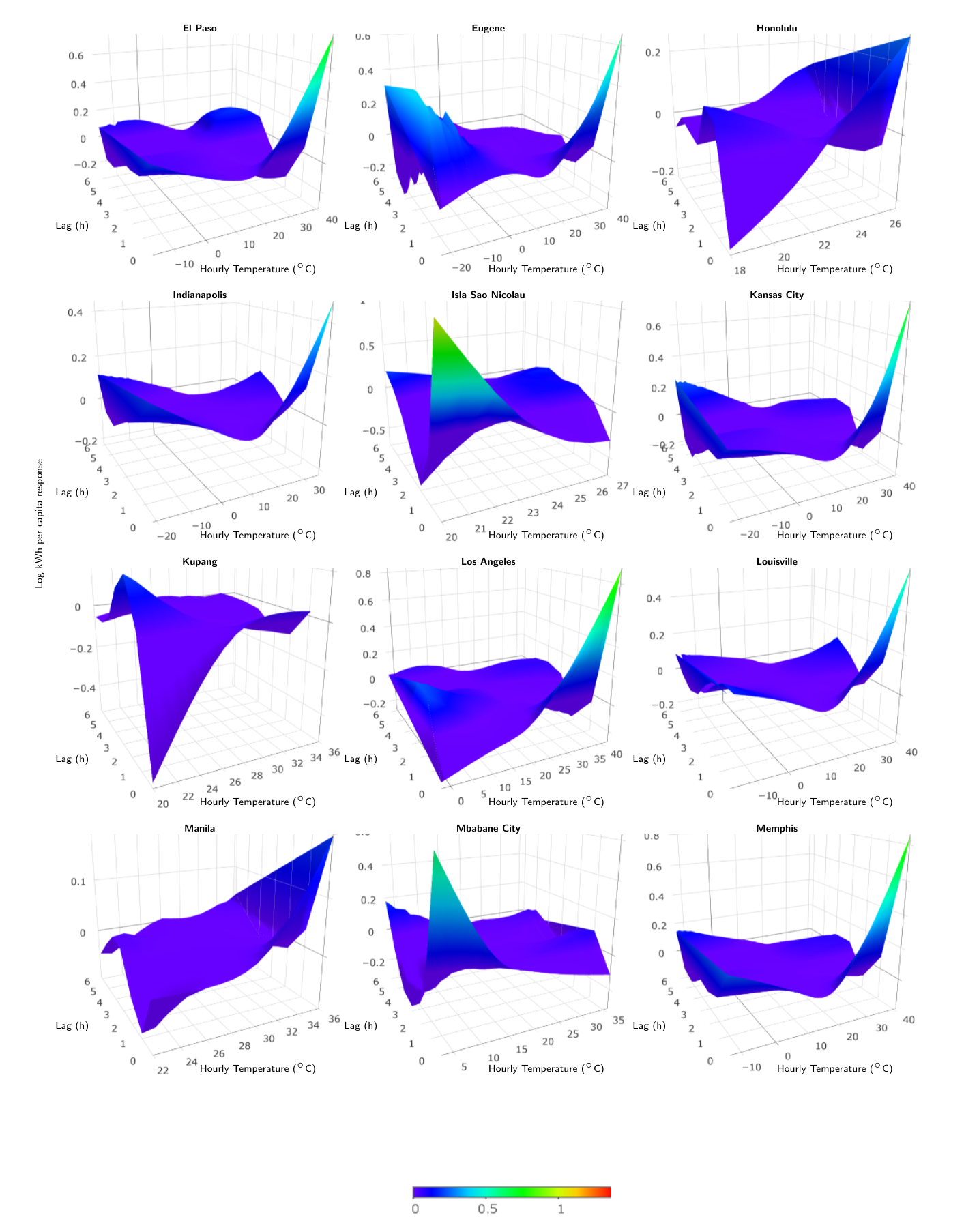


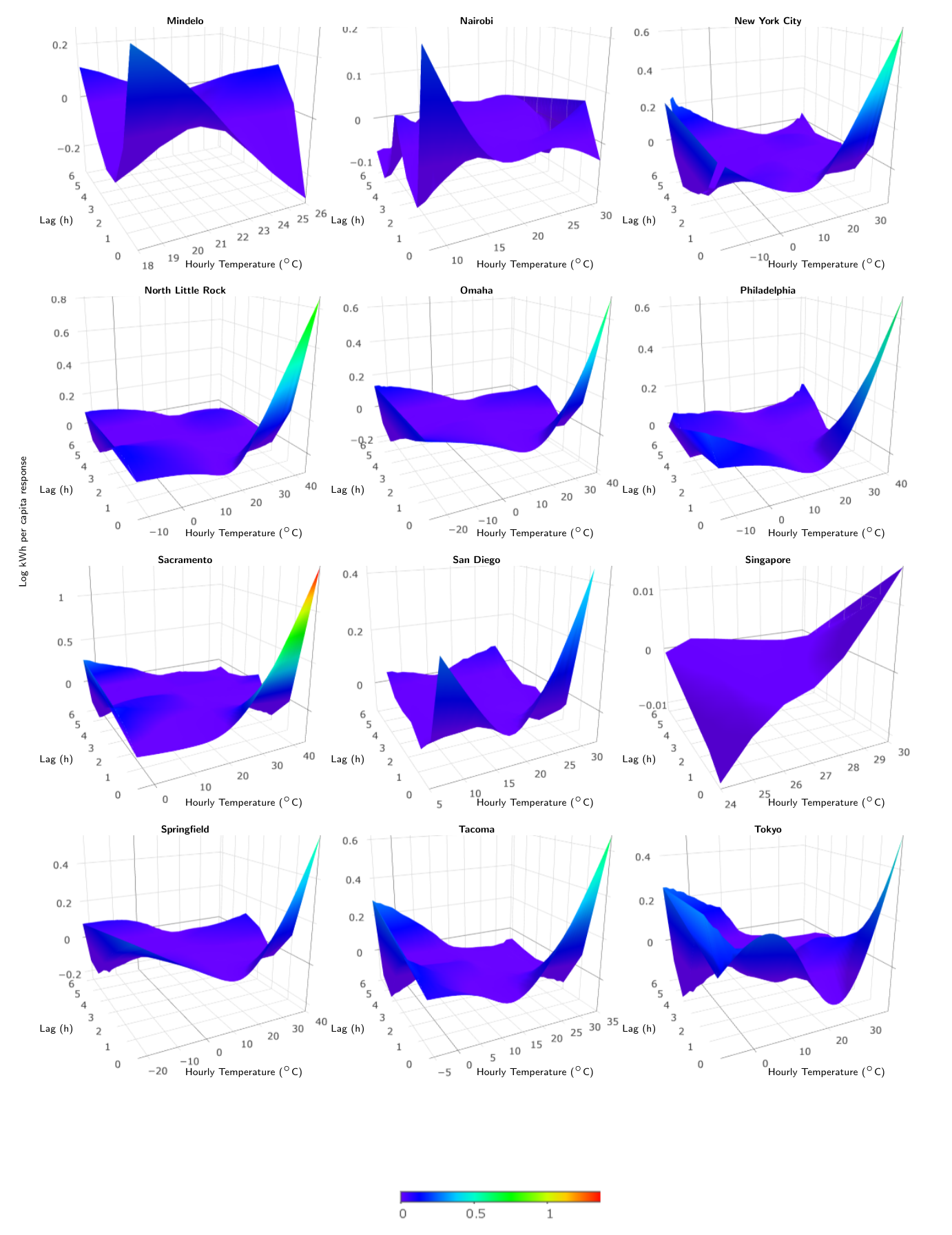
**Figure S1 (continued)**

**Figure S2**


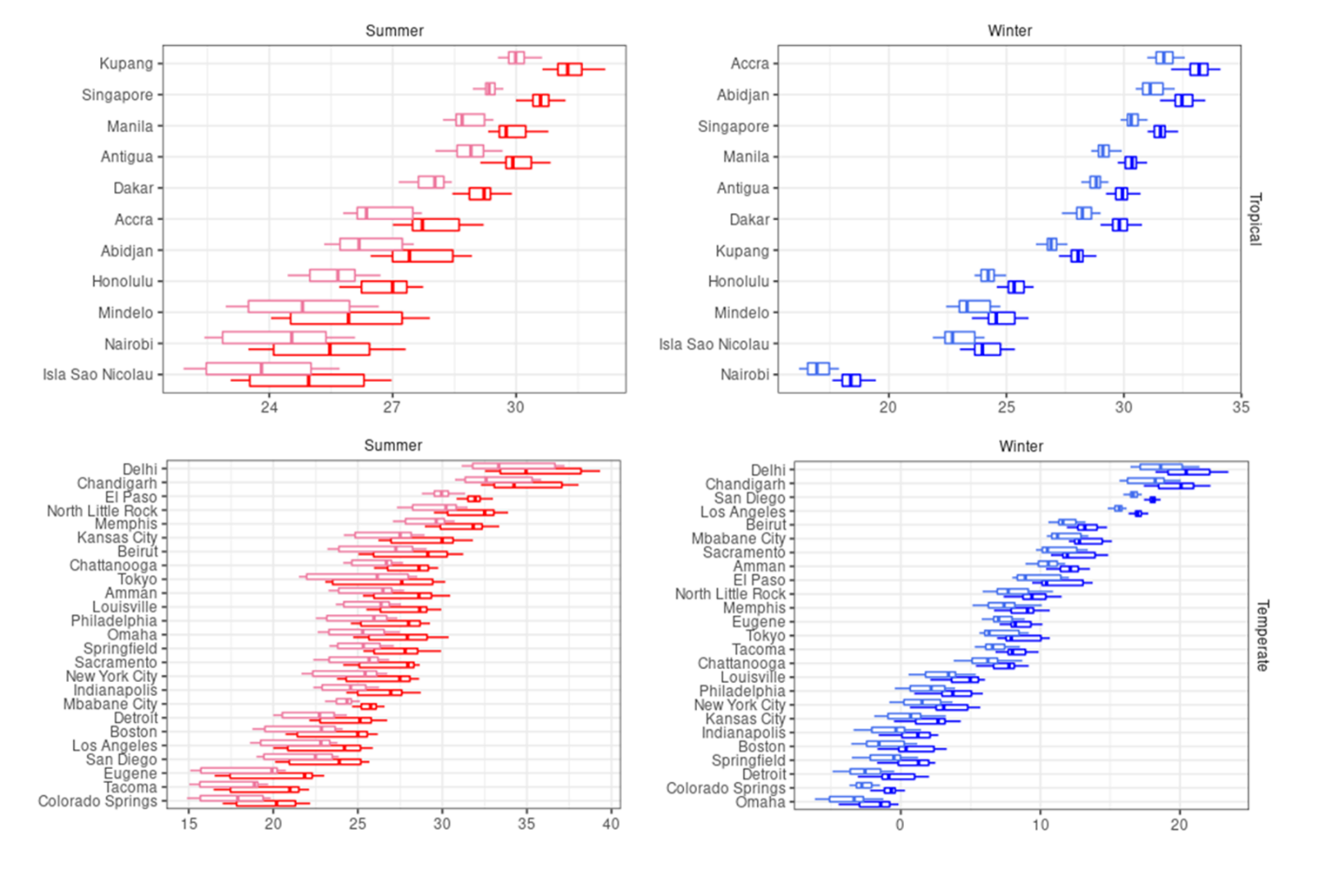
Seasonal temperature distributions (°C) by city for both the historical period and the future under RCP 8.5. Red indicates the period seasonal mean of future summer temperatures, and corresponding light red depicts period seasonal mean of historical summer temperatures. Blue represents the period seasonal mean of future winter temperatures, and light blue indicates the period seasonal mean of historical winter temperatures. Whiskers denote temperatures >25^th^ and <75^th^ percentile of the seasonal mean temperature distribution, box widths represent the interquartile range of these distributions.

**Figure S3**

**
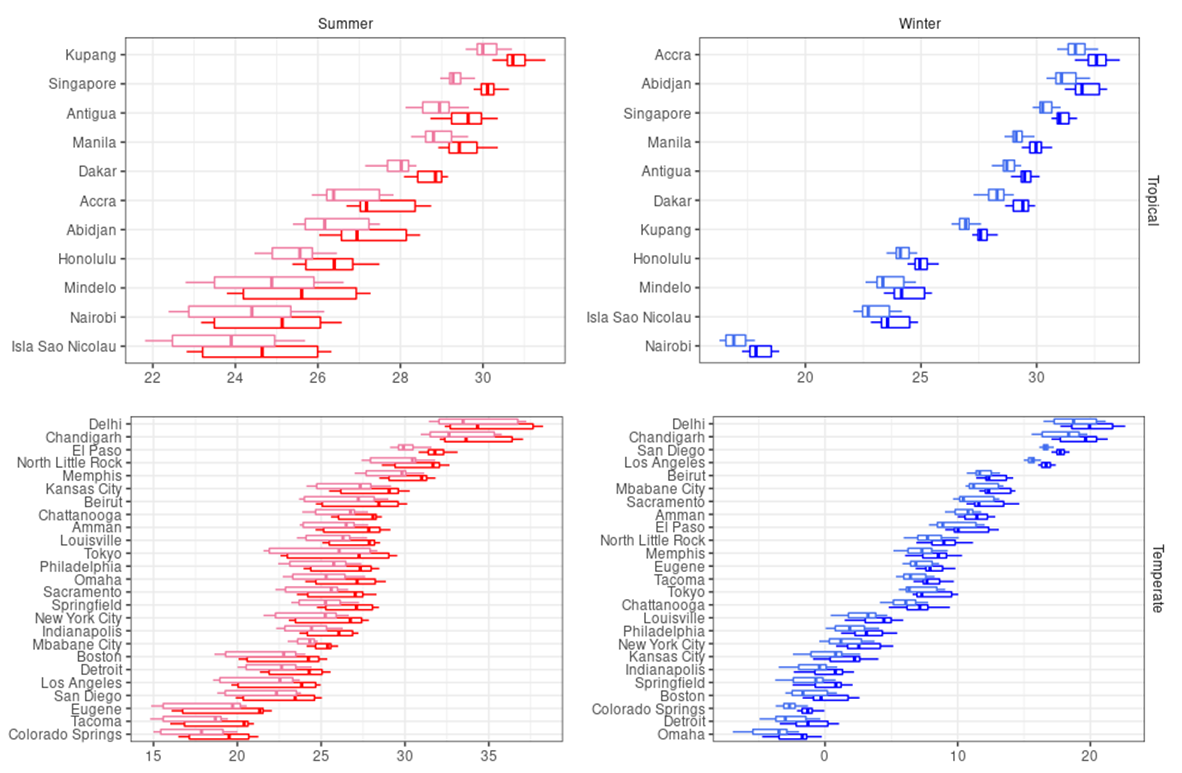
**Seasonal temperature distributions by city for both the historical period and the future under RCP 4.5. Whiskers denote temperatures >25^th^ and <75^th^ percentile of the seasonal mean temperature distribution, box widths represent the interquartile range of these distributions.

**Figure S4**

Median (black), 5^th^ (blue) and 95^th^ (red) per capita percentage change impacts to cities' diurnal load demand profiles under RCP 8.5. 95^th^ percentile for individual GCMs (light red), 5^th^ percentile (light blue).


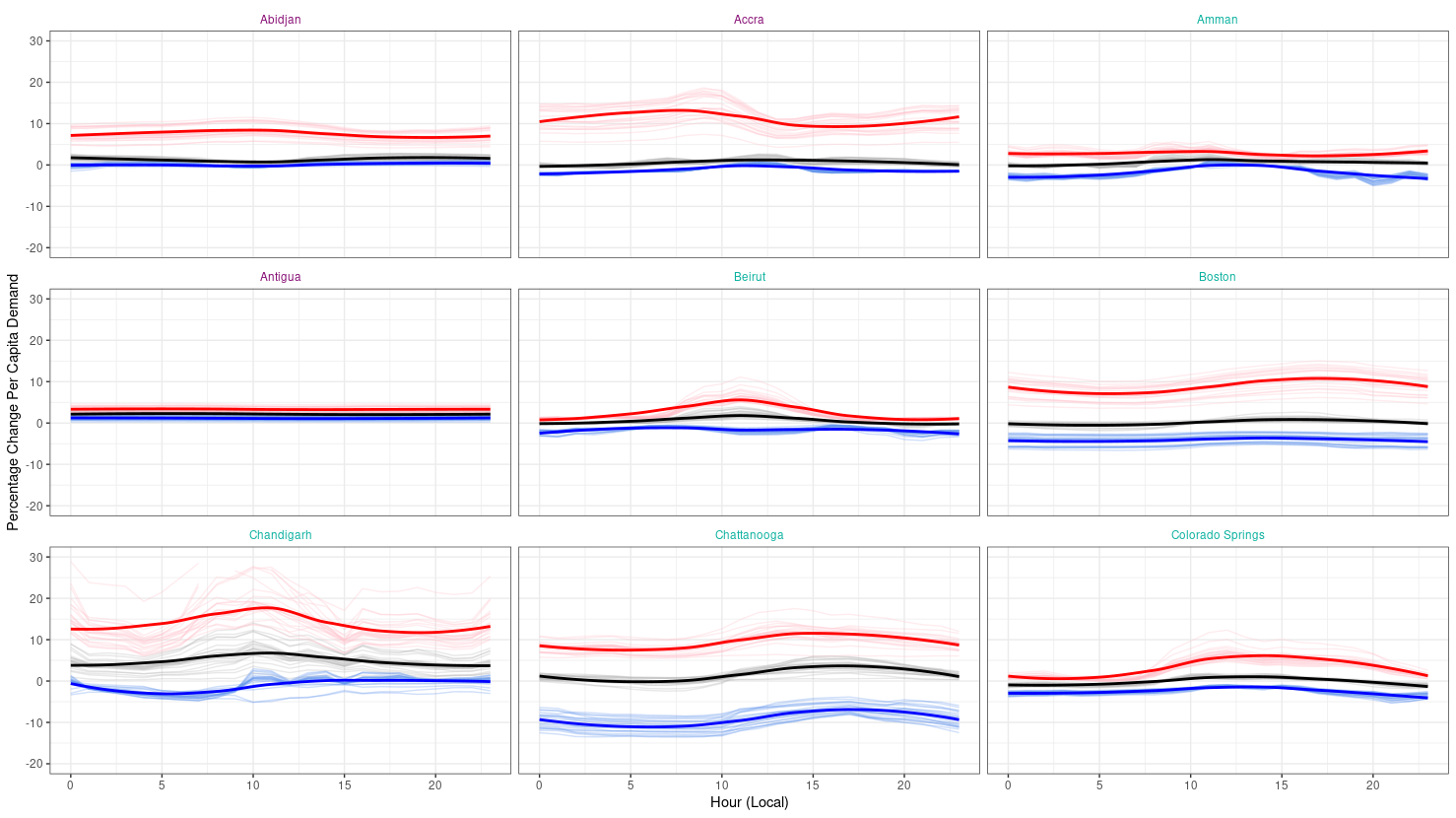

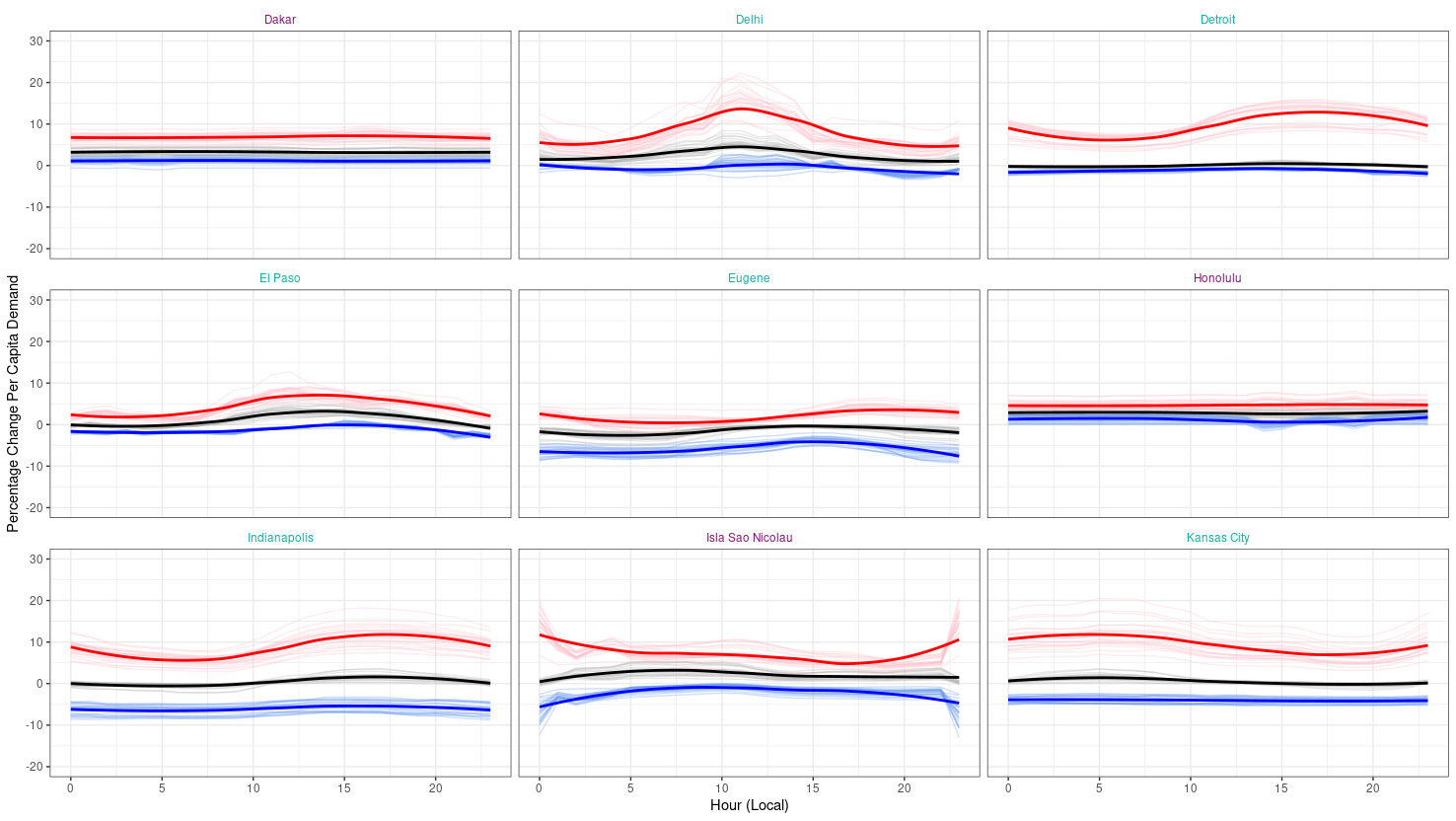


**Figure S4 (continued)**


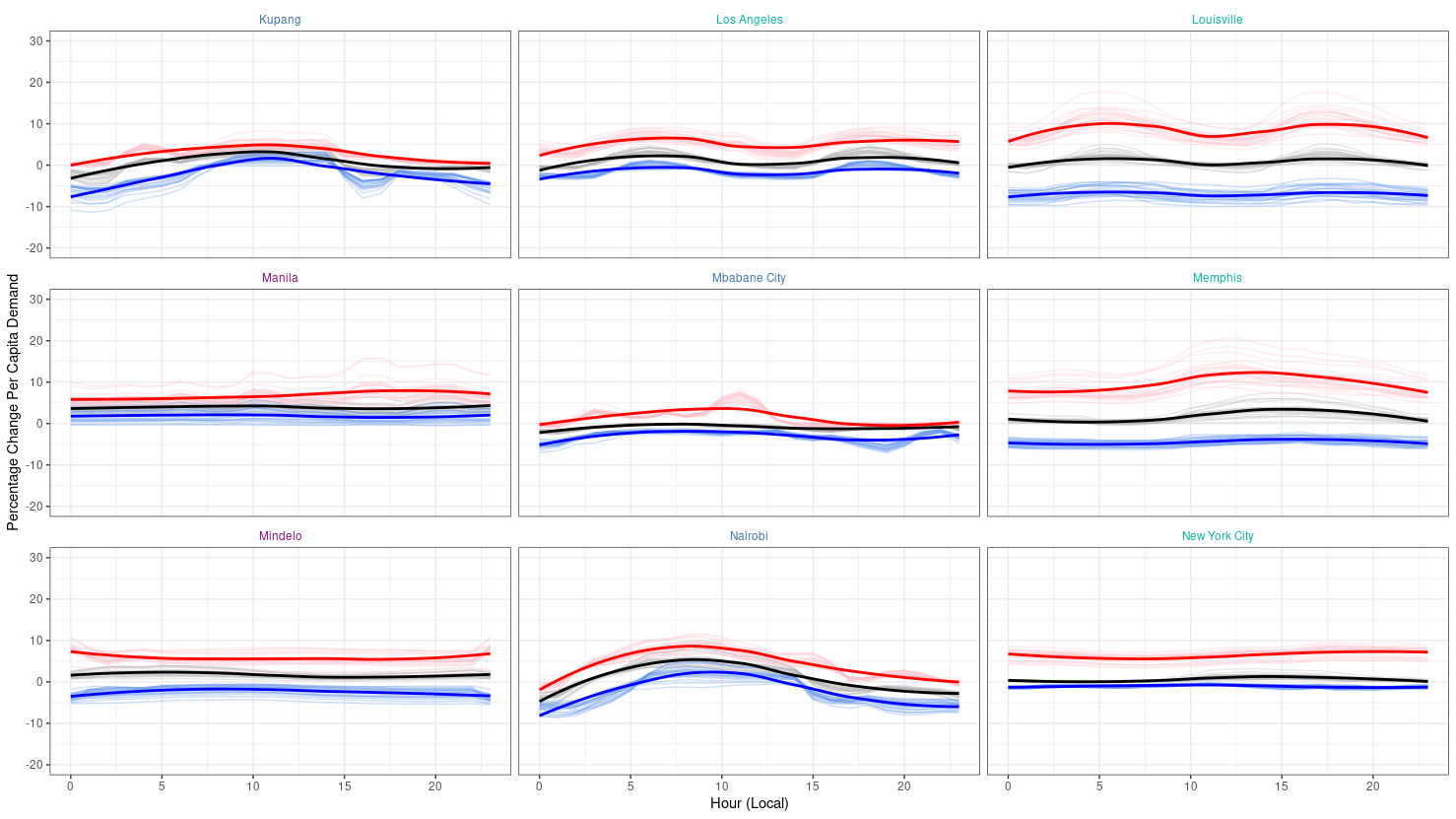

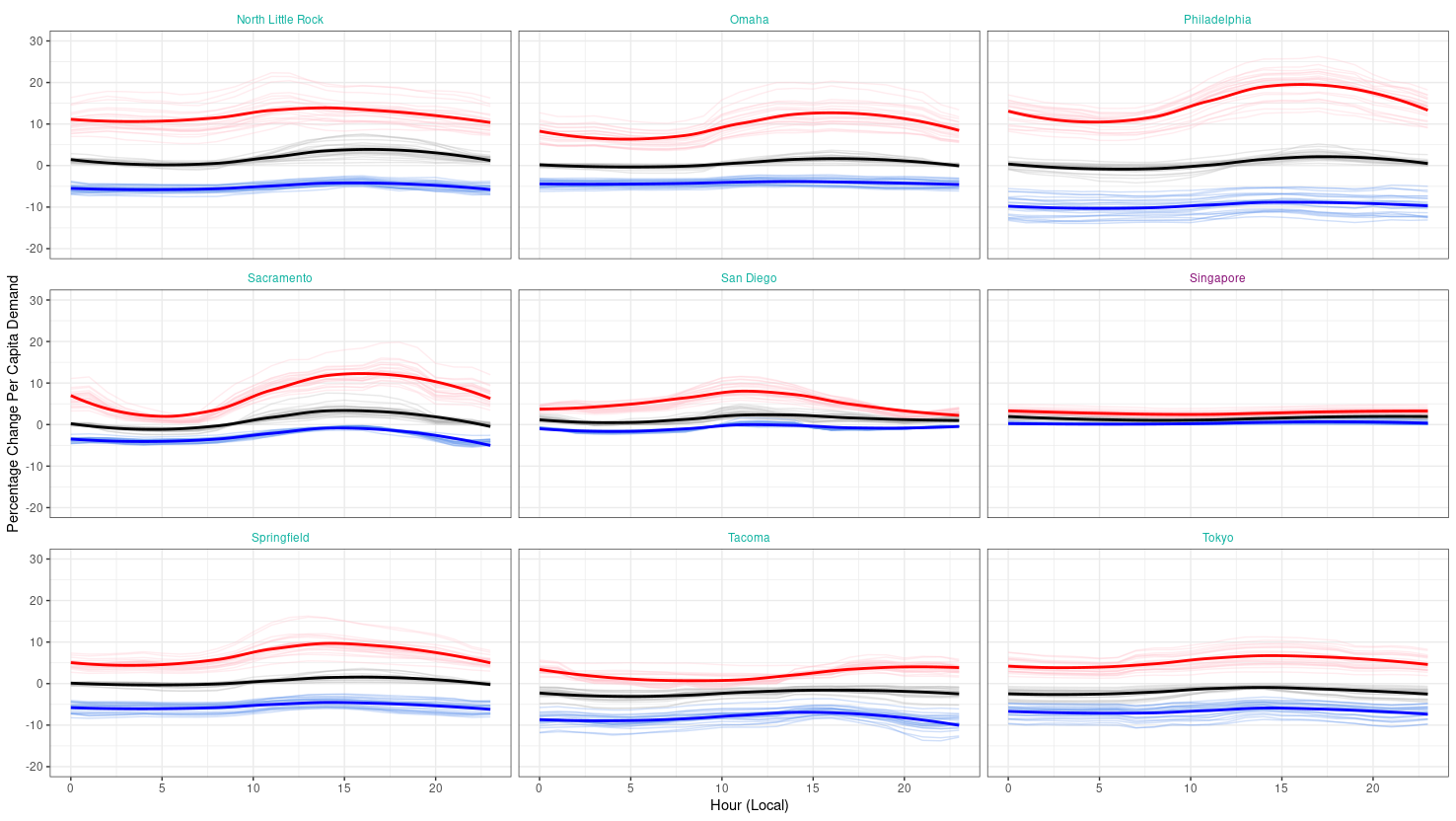


**Figure S5**

Median (black), 5^th^ (blue) and 95^th^ (red) per capita percentage change impacts to cities' diurnal load demand profiles under RCP 4.5


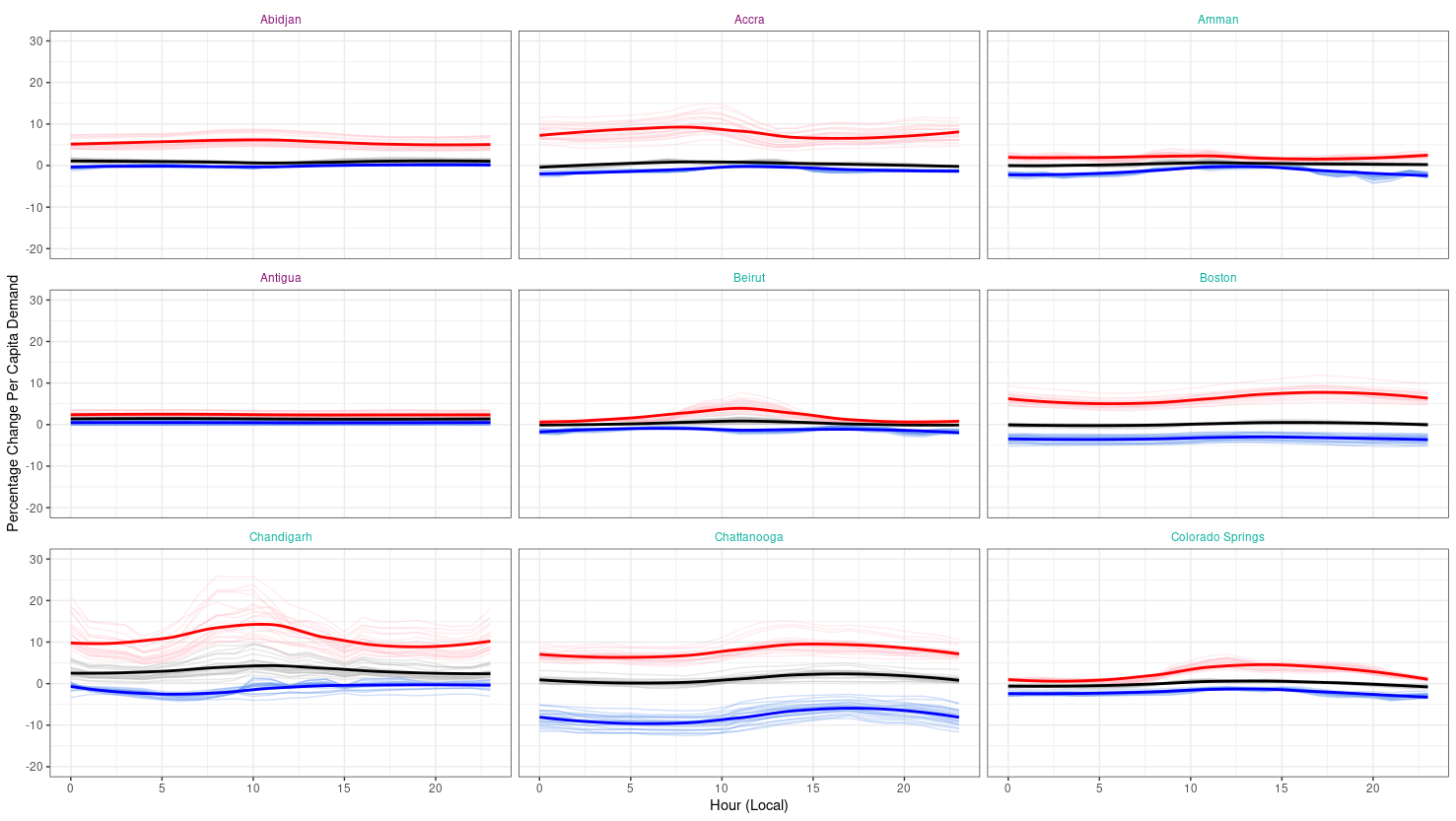

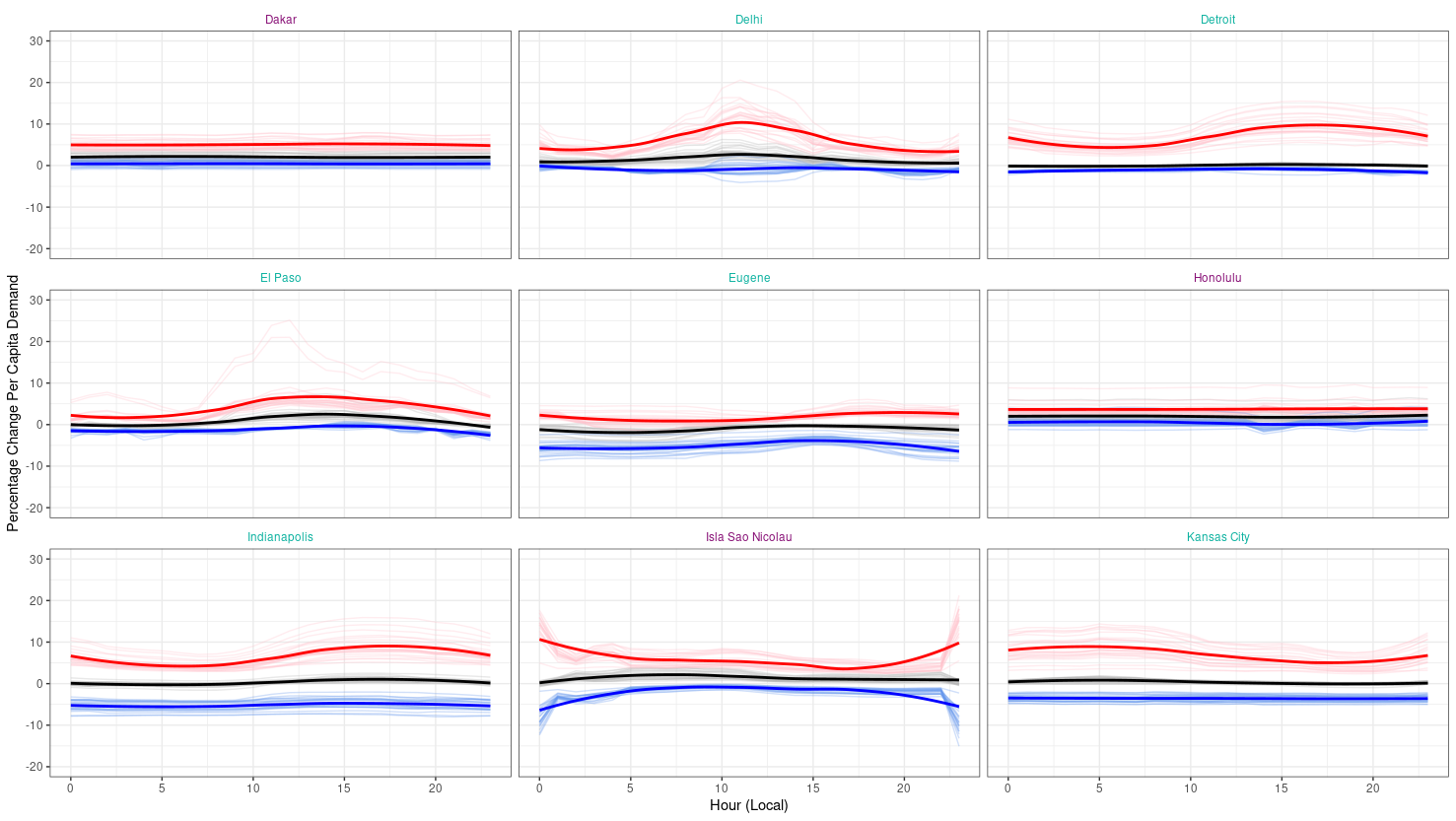


**Figure S5 (continued)**


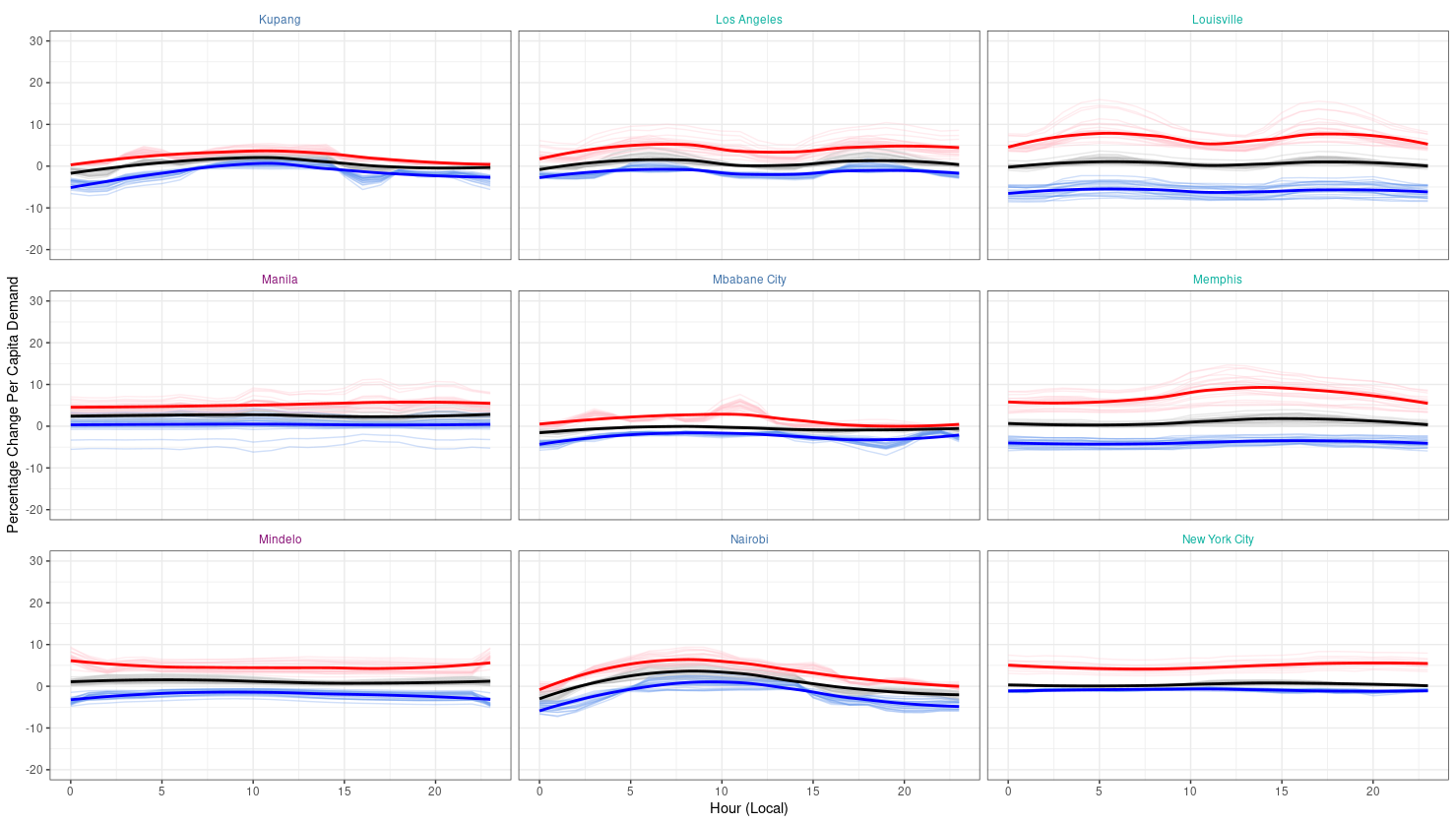

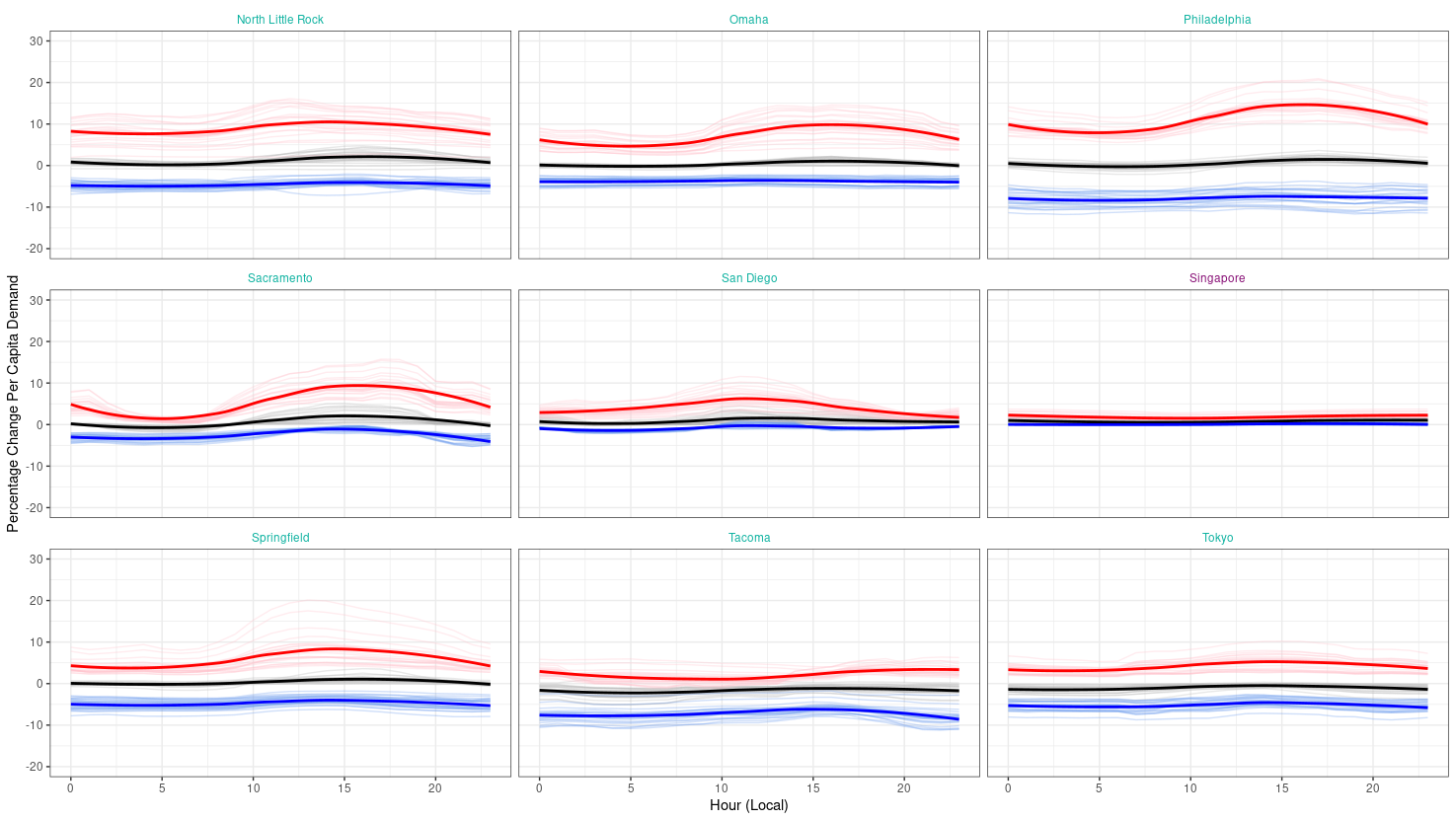


**Figure S6**


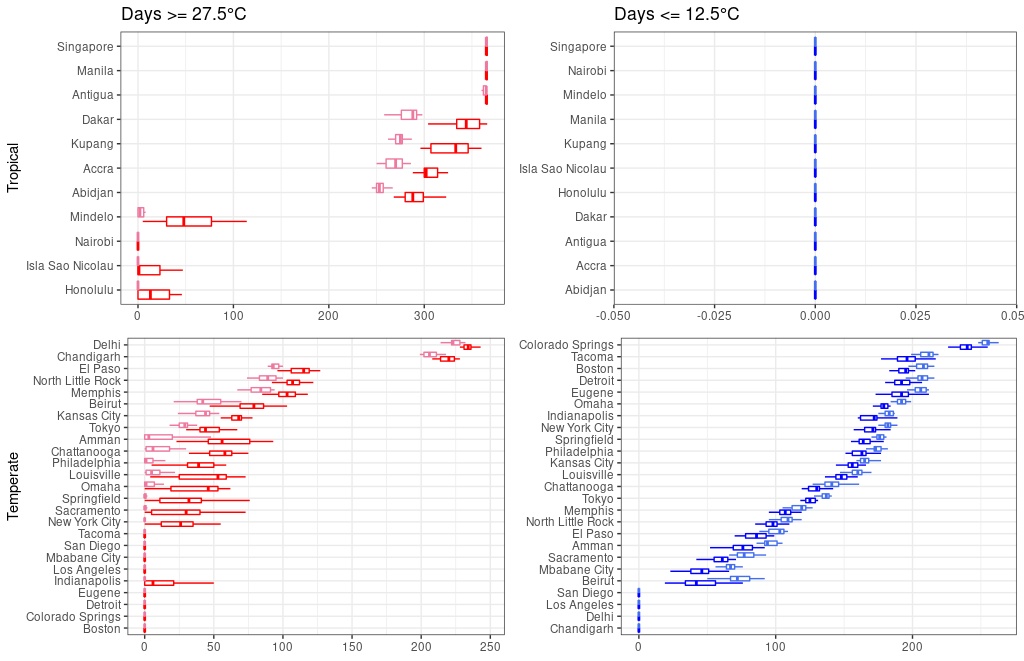
Historical (light colors) and projected (dark colors, RCP 8.5) hot days (≥ 27.5°C, red) and cold days (≤12.5°C, blue). Whiskers denote temperatures >25^th^ and <75^th^ percentile of the seasonal mean temperature distribution, box widths represent the interquartile range of these distributions.

**Figure S7**

**
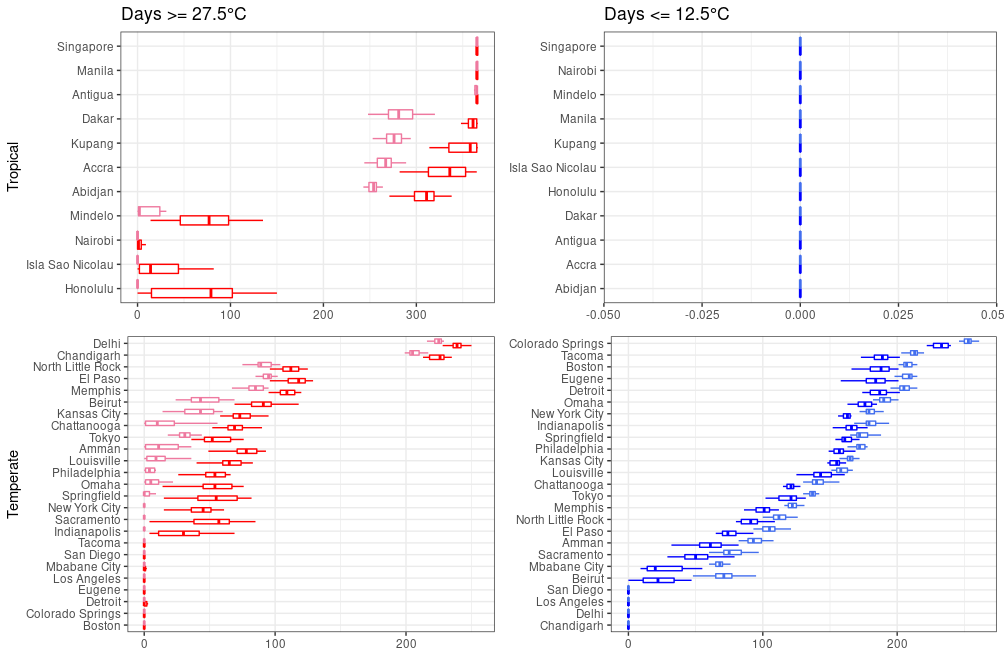
**Historical (light colors) and projected (dark colors, RCP 4.5) hot days (≥ 27.5°C, red) and cold days (≤12.5°C, blue). Whiskers denote temperatures >25^th^ and <75^th^ percentile of the seasonal mean temperature distribution, box widths represent the interquartile range of these distributions.

**Figure S8**

**
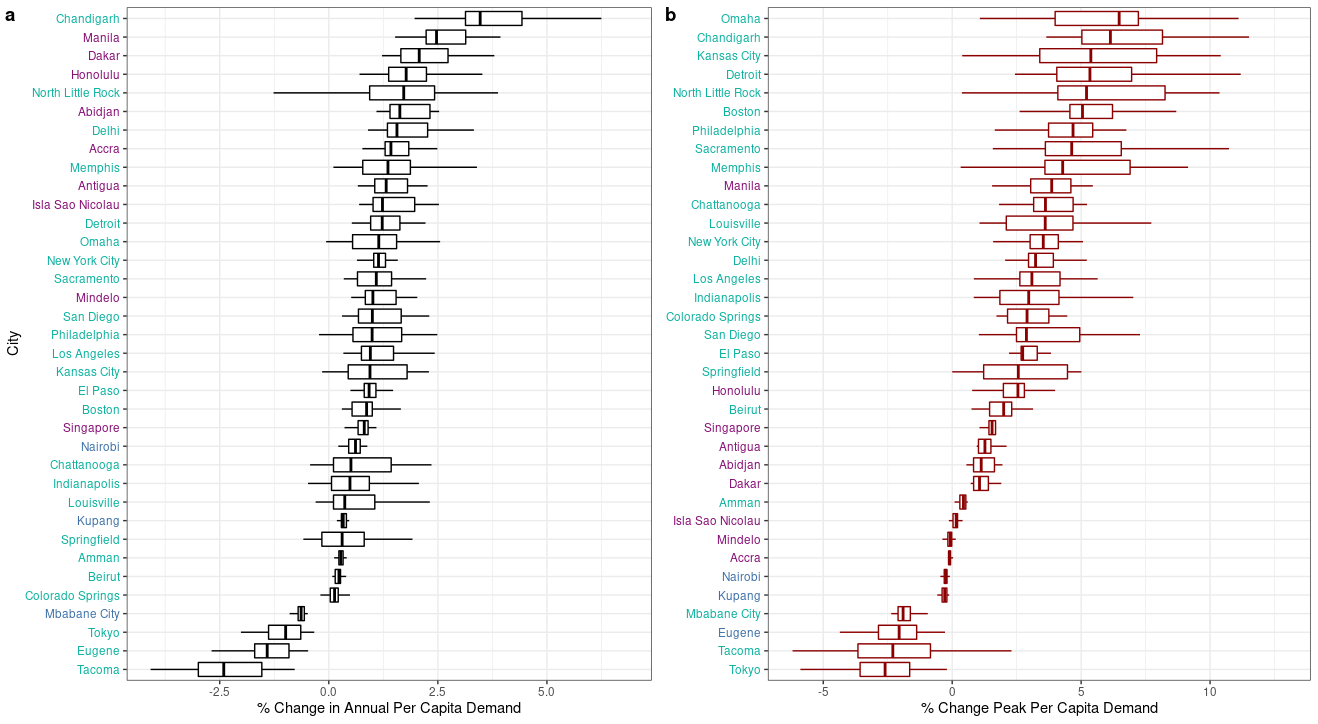
**Projected shifts in the percentage change of per capita demand across GCMs under RCP 4.5 (a - annual, b - 95^th^ percentile)

**Figure S9**

Influence of contemporaneous (lag 0) versus lagged (lags1-6) peak temperatures on mean demand in tropical (left) and temperate(right) cities. Gray circles depict mean MWh per capita demand for peak temperatures during the historical period, squares depict the median multi-model mean MWh per capita demand for peak temperatures in 2050 under RCP 4.5, arrows indicate direction of change.


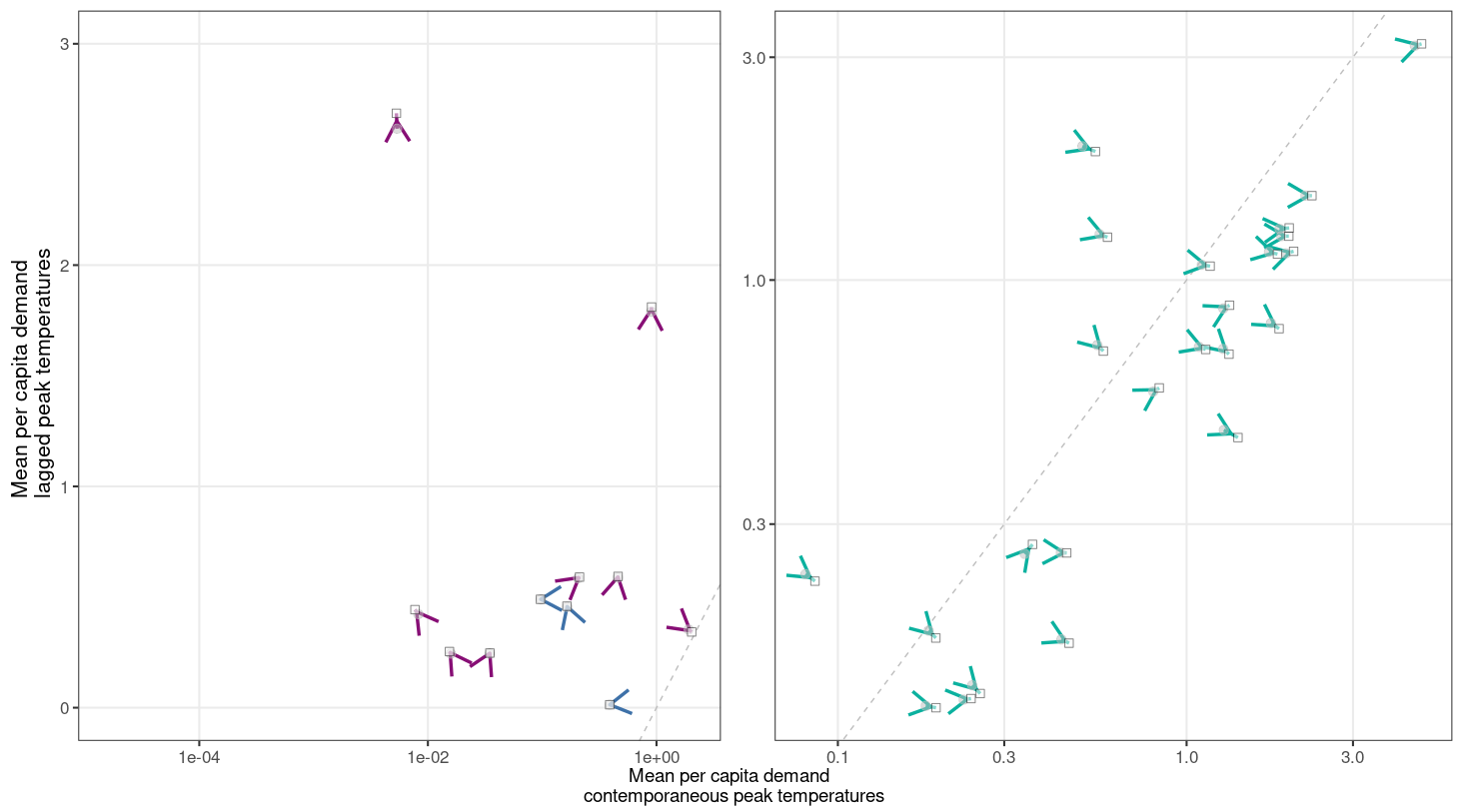


**Figure S10**

Historical and projected load duration curves (LDCs) under RCP 8.5 (continued from Figure 6 in the main text). Historical electricity demand is shown in blue, projected median demand under RCP 8.5 in red, individual GCMs under RCP 8.5 in yellow. Bottom panels illustrate seasonal shifts in demand under historical and future scenarios where blue-winter, red-summer, spring-green, and fall-orange. Bottom set of panels represent historical demand, top set of panels represent demand under RCP 8.5

**
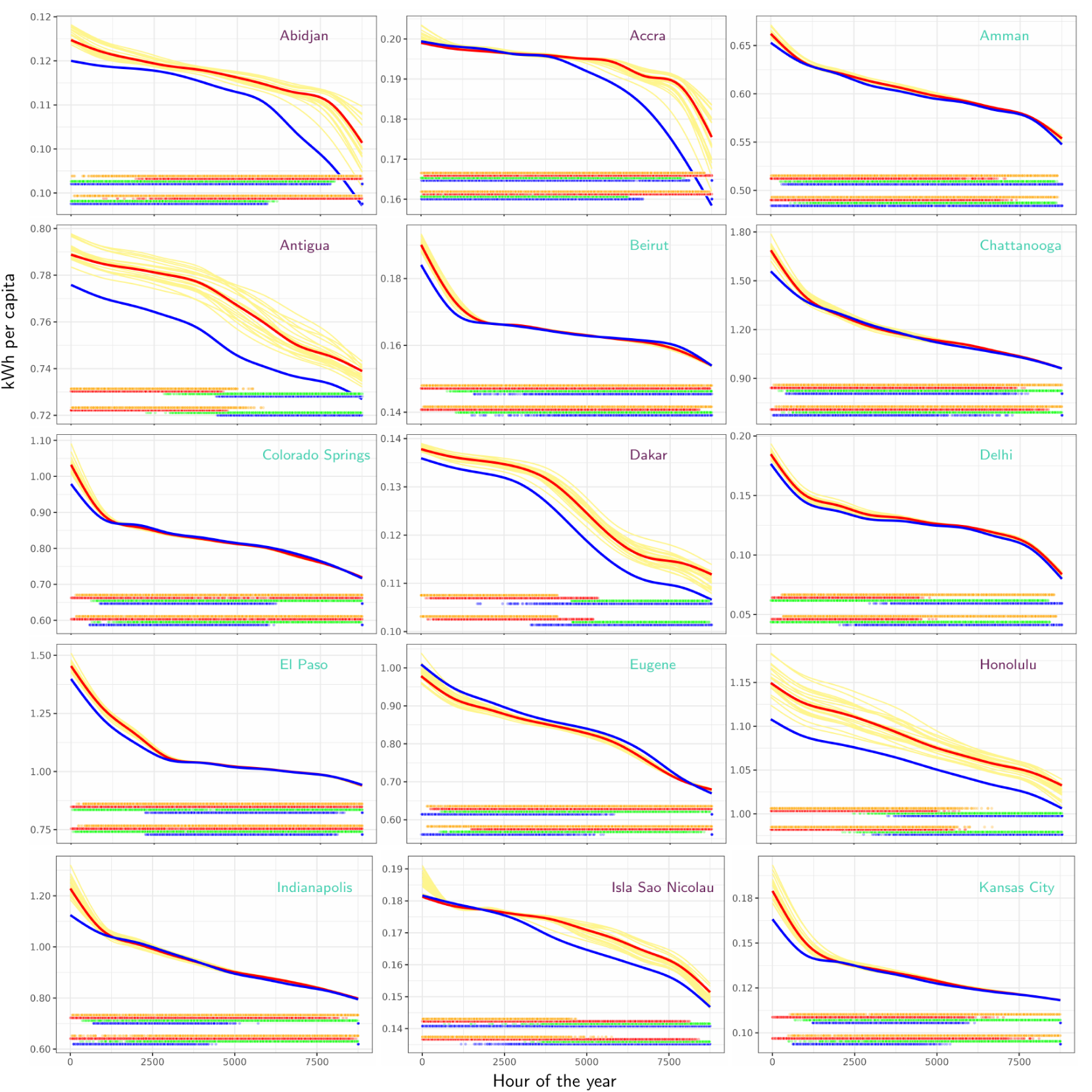
**

**Figure S10 (continued)**

**
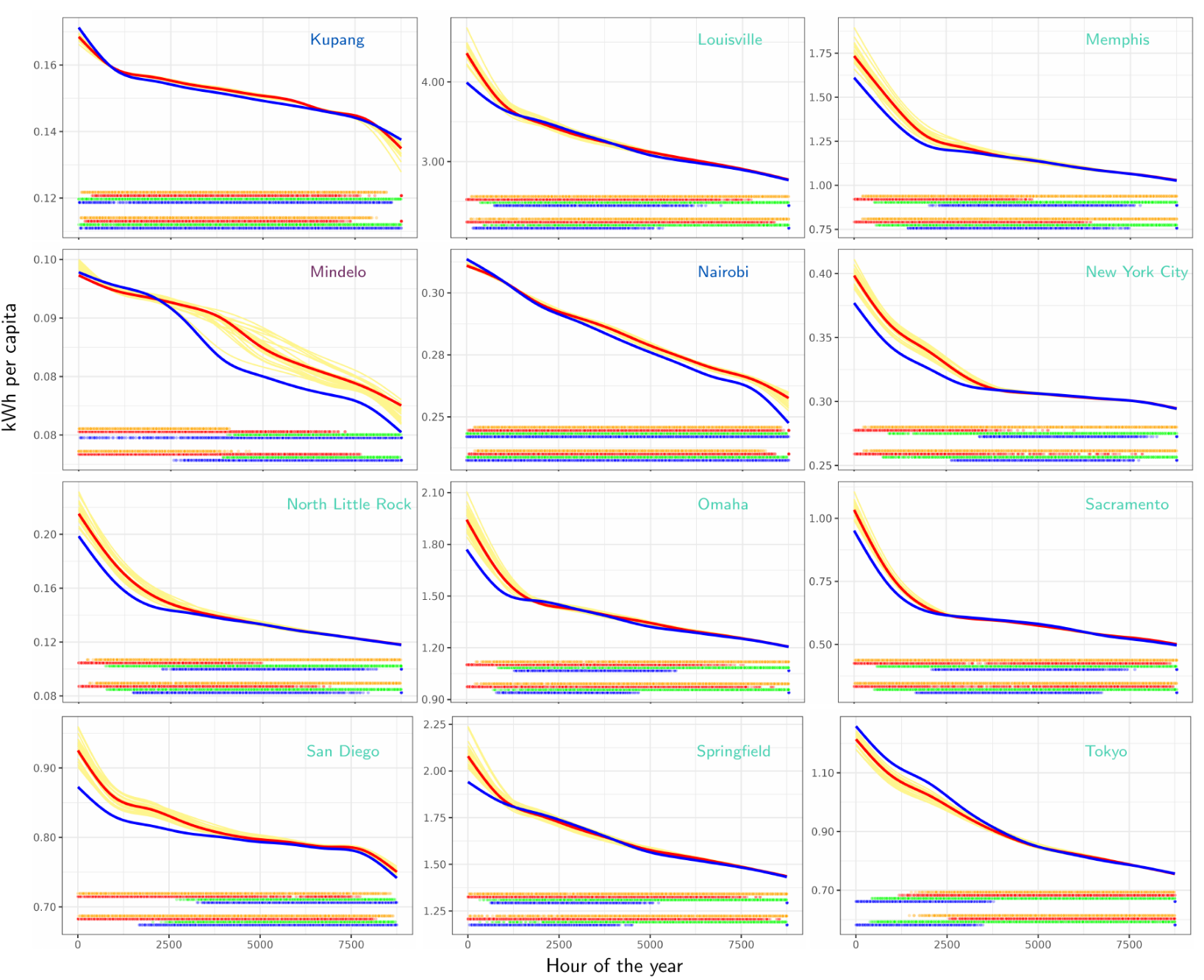
**

**Figure S11**


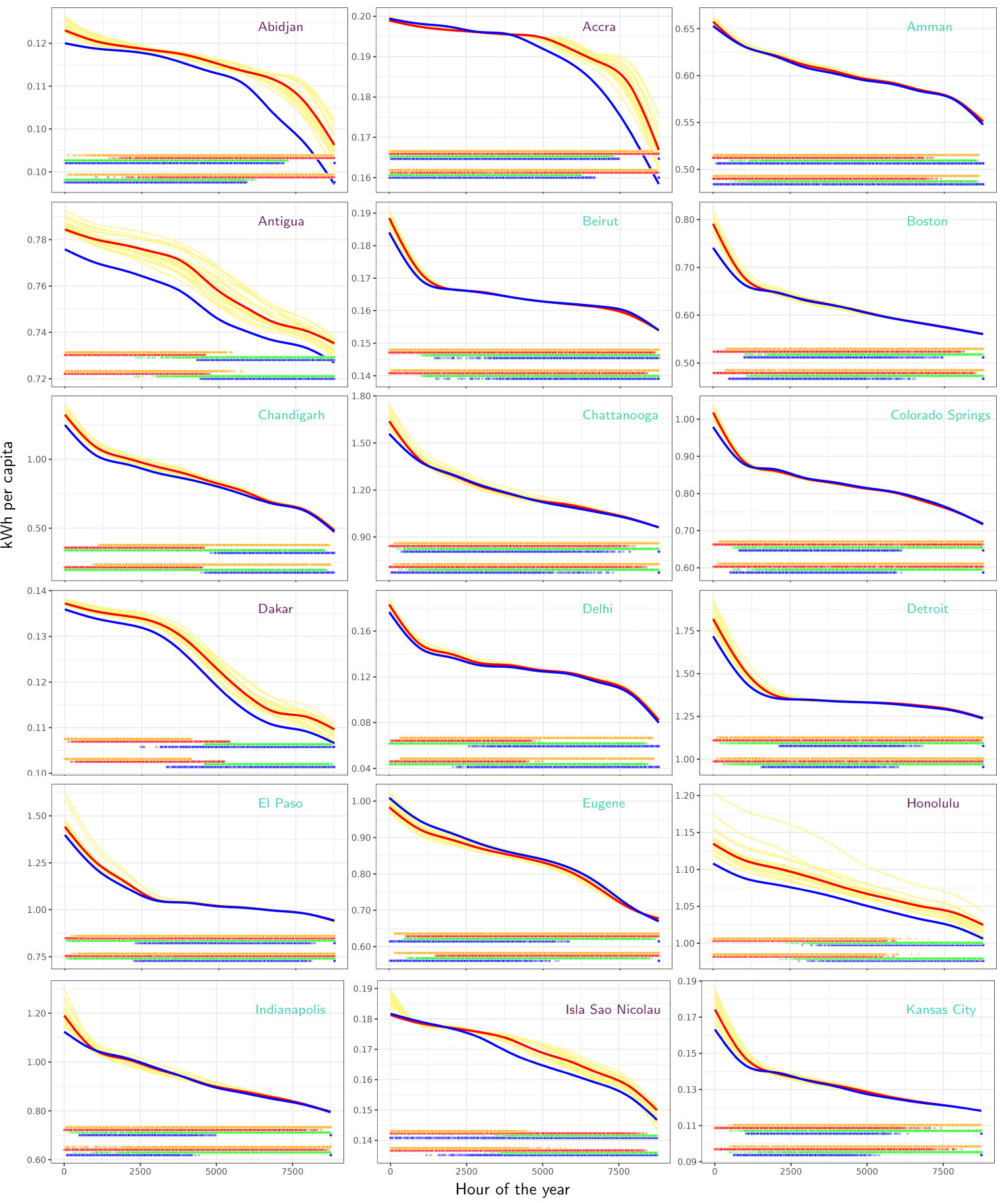
Historical and projected load duration curves (LDCs). Historical electricity demand is shown in blue, projected median demand under RCP 4.5 in red, individual GCMs under RCP 4.5 in yellow. Bottom panels illustrate seasonal shifts in demand under historical and future scenarios where blue-winter, red-summer, spring-green, and fall-orange. Bottom set of panels represent historical demand, top set of panels represent demand under RCP 4.5.

**Figure S11 (continued)**

**
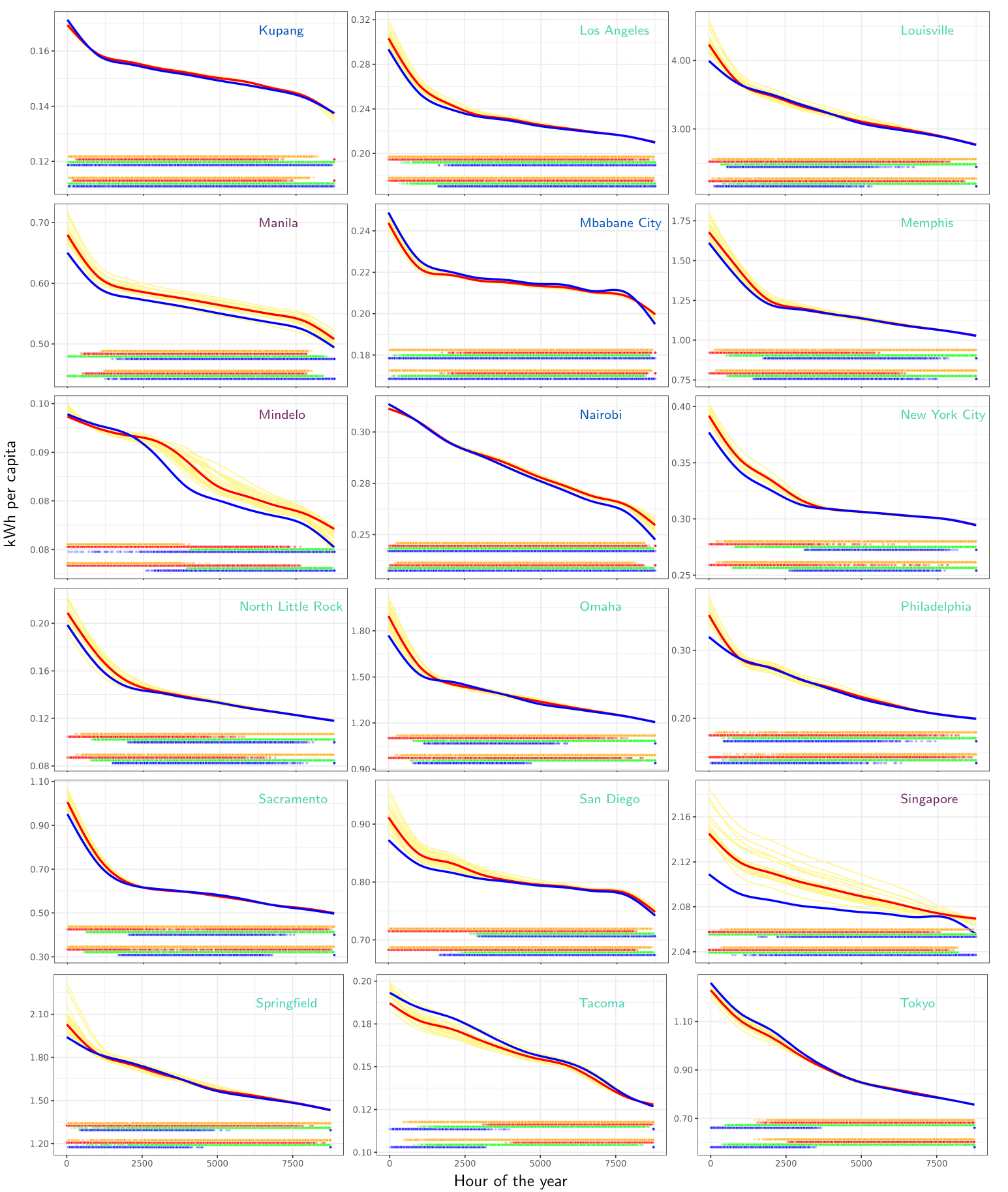
**

**Table S2**

Impact to load levels (GWh) under RCP 4.5 for all temperatures (T), T≥ set-point temperatures (T*), and T<T*


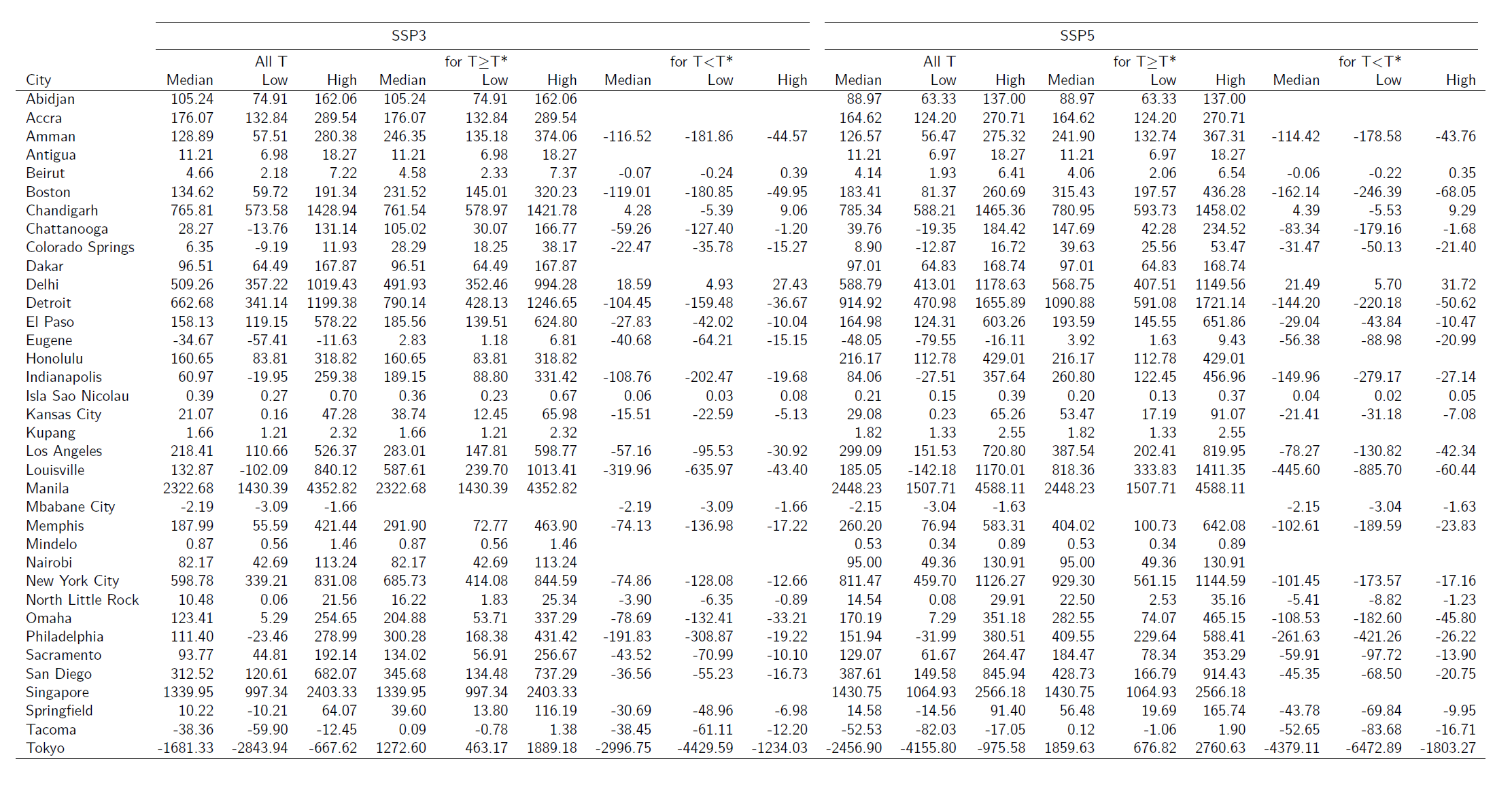
**Figure S12**

**
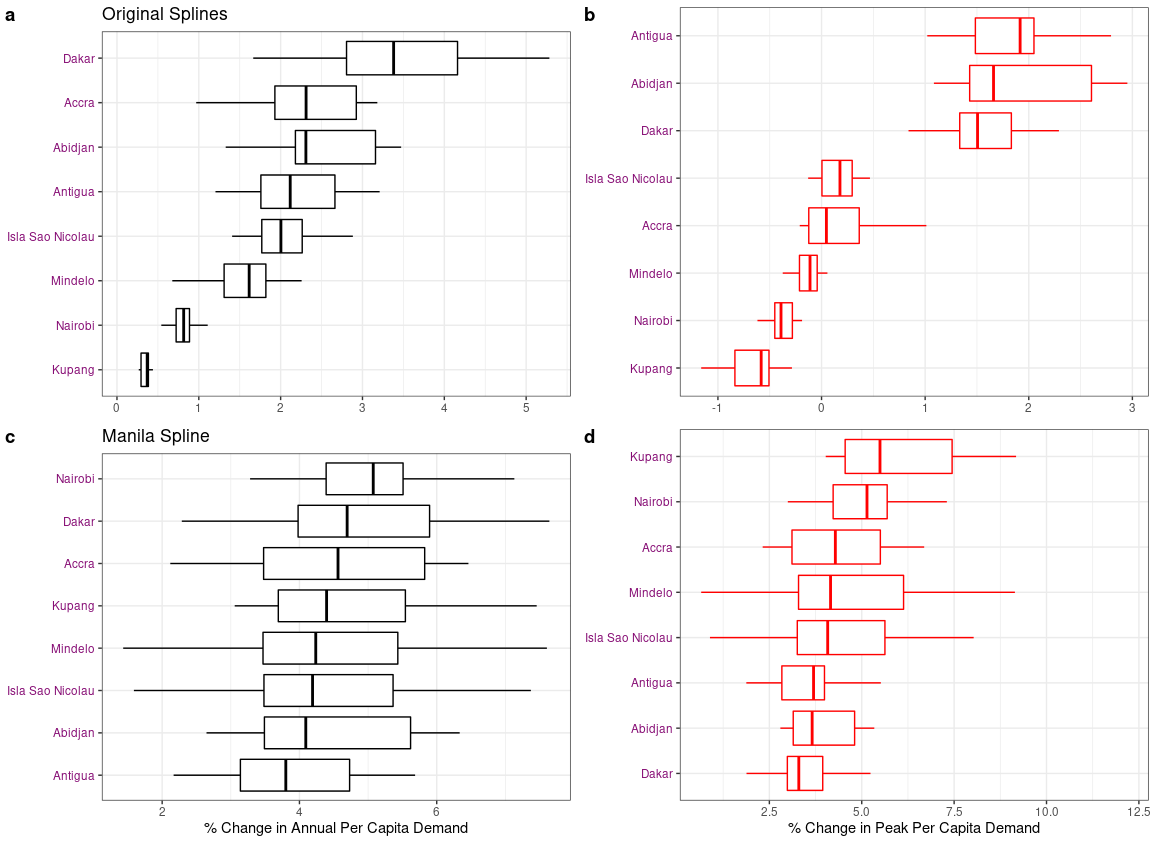
**Percentage change of per capita demand across GCMs under RCP 8.5 for tropical cities—comparison of annual (a) and peak (b) demand shifts with tropical cities’ original demand-response functions to annual (c) and peak (d) shifts when endowed with Manila’s demand-response function.

**Table S3**

Impact to load levels (GWh) under RCP 8.5 for all temperatures (T), T≥ set-point temperatures (T*), and T<T*-comparison of impacts for tropical cities with original demand-response functions (top) to shifts when endowed with Manila’s demand-response function (bottom).

**
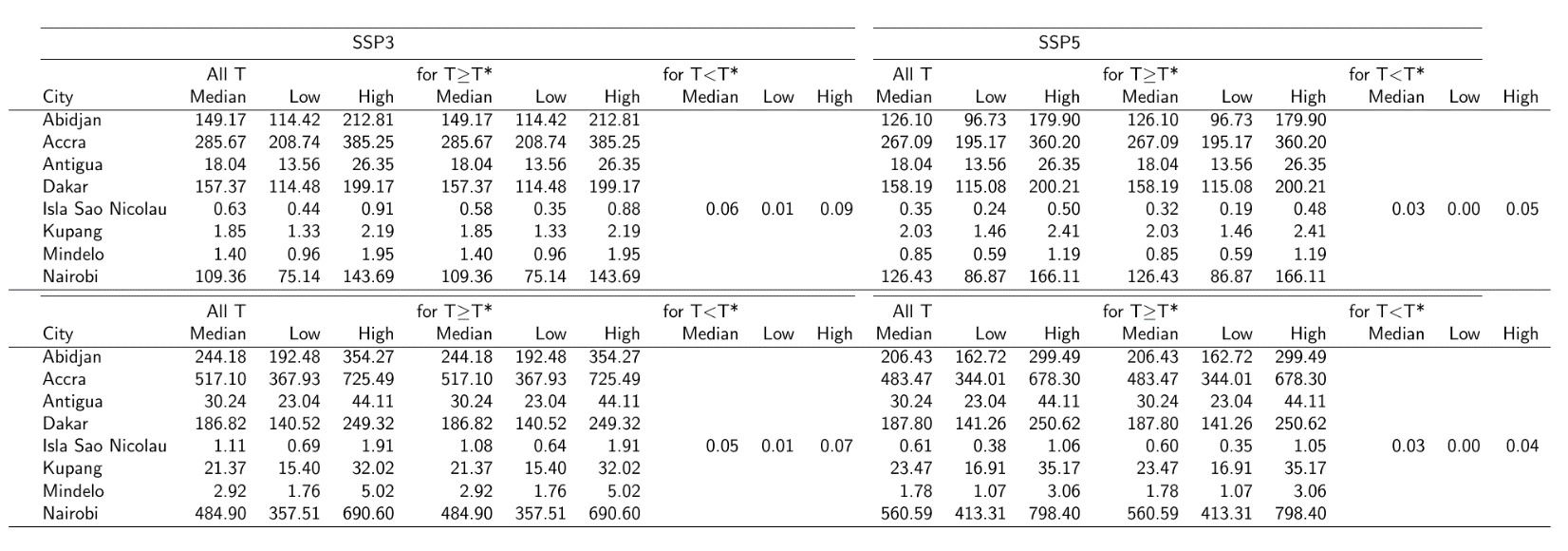
**

**RCP 4.5 Discussion**

In contrast to the high emissions scenario posited by RCP 8.5, RCP 4.5 describes a scenario of stabilization in which radiative forcing is maintained through a range of greenhouse gas (GHG) emissions reduction technologies and strategies. Under RCP 4.5, cities experience smaller shifts in their temperature distributions as compared to RCP 8.5—1-3°C in tropical cities, 3-5°C in temperate cities—resulting in modest impacts to net (-2.4% to 3.7%) and peak (-2.5% to 7%) per capita change in electricity demand across our sample of cities. Peak demand impacts are concentrated in mid-latitude temperate, largely North American, cities as observed under RCP 8.5. Thus, while the magnitude of impacts to electricity demand are less pronounced under more moderate warming, the pattern of incidence remains similar between RCPs, consistent with other empirical studies of this kind (van Ruijven et al, 2019; Wenz et al, 2017; Aufhammer and Aroonruengsawat, 2011). These results reinforce our findings that heterogeneity in the structure of electricity demand play an important role in the effects of future warming on demand in tropical cities; and moreover, that patterns of previous demand responses estimated for developed, temperate cities do not echo the pattern of temperature dependence of electricity consumption in developing, tropical areas.

However, we note that these findings assume that the shape and structure of electricity demand, in addition to the current fuel mix of energy supply for heating and cooling, will remain constant to mid-century. The stabilization of radiative forcing under RCP 4.5 presumes the deployment of a variety of emissions mitigation strategies and technologies that are not captured using our methodology. Many countries and cities have proposed mitigation and adaptation plans that include specific energy system goals and objectives, but how, and which, of these policies will be implemented across diverse urban areas remains uncertain. There are a variety of ways in which these decisions impacting electricity generation, transmission, and capacity will ultimately manifest in the structure of electricity demand (e.g., Steinberg et al, 2020). A number of cities have set forth or are already implementing renewable energy policies (Hess and Gentry, 2019; Murdock et al, 2019), the resulting diversification of energy supply through the adoption of largely renewable-based electricity generation will require modification of the energy system which is likely to influence the shape of a cities’ demand response surface. Cities are also exploring initiatives to electrify urban transportation systems and substitute electricity for natural gas in the building sector.^^[[1]](#footnote-1)^^ However, the trend toward electrification indicates a conflict over the need to actively facilitate adaptation to climate change impacts while reducing GHG emissions to mitigate further warming. If such mitigation measures increase cities’ baseline electric power consumption, but their populations’ attempts to adapt to increasing temperature extremes via more intensive air conditioner utilization amplify total—and particularly peak—demand for electricity, then baseline capacity projections for generation, transmission and distribution infrastructure may be inadequate. Moreover, depending on the mix of fuels used to generate additional electricity in times of peak temperature-driven demand, consumption-driven GHG emissions in cities may actually *increase*, creating conflict between adaptation to heat extremes and mitigation goals. A further caveat to note is the added interaction of demographic shifts in population and urbanization in tandem with a mitigation-based energy system which may additionally modify the structure of weather-sensitive demand, and thus projections of mid-century demand under RCP 4.5.

**Methods and Data**

*Theoretical basis for empirical approach*

The following discussion draws heavily on Rabl (1988). A widely used, conceptually simple method for analyzing building energy use is thermal networks, the thermodynamic analogue of electrical circuits that exhibit dynamic behavior. The simplest network structure that exhibits dynamics is the 1R1C model (Fig. S13), so named because it combines one impedance (resistance to heat flow, indicated by $R$, the inverse of the total heat loss coefficient) and one capacitance (thermal energy storage, indicated by $C$). Both $R$ and $C$ are functions of the building envelope’s material properties and geometry. The dynamics of the system are driven by three time-varying forcings that provide heat input: exogenous outdoor temperature ($T_{O}$) that determines conduction of heat through the building shell, solar energy flux ($F_{S}$) that determines the building shell’s radiative absorption or emission, and internal loads ($F_{N}$) principally heat given off from occupants’ bodies or appliances that they use. The endogenous variable that is of particular interest is the (negative) summer cooling load from air conditioning ($F_{A}$), which functions to maintain the internal temperature ($T_{I}$) within a comfortable range. The 1R1C model is a purely expository, brutal simplification of reality. Its strongest assumption is lumped heat capacity at the building interior (as opposed to a shell of high heat capacity materials such as brick or concrete), which constrains the interior air, objects and surfaces to a common thermal mass temperature, $T_{C}$, at each instant of time. More realistic models relax this structure at the cost of a substantial increase in complexity, e.g., the five resistance one capacitance (5R1C) network based on the ISO 13790 standard (see Jayathissa et al, 2017).

**Figure S13**

1R1C Thermal Network for a Building with Space Conditioning

$$F_{N}-F_{A}$$

$$F_{S}$$

$$T_{I}=T_{C}$$

$$T_{O}$$

$$R$$

$$C$$

Using $t$ to index time, instantaneous energy balance for the 1R1C network is given by

$F_{S}\left( t \right)+F_{N}\left( t \right)-F_{A}\left( t \right)=\frac{1}{R}\left( T_{I}\left( t \right)-T_{O}\left( t \right) \right)+c\dot{T}_{I}\left( t \right).$ (S.1)

Integrating this expression yields

$\int_{0}^{\infty} \left[ a_{I}\left( \tau\right)T_{I}\left( t-\tau\right)-a_{O}\left( \tau\right)T_{O}\left( t-\tau\right)-a_{S}F_{S}\left( t-\tau\right)-a_{N}F_{N}\left( t-\tau\right)+a_{A}F_{A}\left( t-\tau\right) \right]d\tau=0$ (S.2)

in which $a_{I}$, $a_{O}$, $a_{S}$, $a_{N}$ and $a_{A}$ are transfer functions that depend only on the properties of the building under consideration. Each term in eq. (S.2) can be expressed as $\int_{0}^{\infty} a\left( \tau\right)u\left( t-\tau\right)d\tau=\Sigma_{n=0}^{\infty}\alpha_{n}\frac{\partial^{n}u\left( t \right)}{\partial t^{n}}$, with coefficients $\alpha_{n}=\frac{1}{n!}\int_{0}^{\infty} \left( -\tau\right)^{n}a\left( \tau\right)d\tau$, enabling the dynamics of energy use and temperature to be approximated by the differential equation:

$$\alpha_{I,0}T_{I}\left( t \right)-\alpha_{O,0}T_{O}\left( t \right)-\alpha_{S,0}F_{S}\left( t \right)-\alpha_{N,0}F_{N}\left( t \right)+\alpha_{A,0}F_{A}\left( t \right)$$

$$+\alpha_{I,1}\dot{T}_{I}\left( t \right)-\alpha_{O,1}\dot{T}_{O}\left( t \right)-\alpha_{S,1}\dot{F}_{S}\left( t \right)-\alpha_{N,1}\dot{F}_{N}\left( t \right)+\alpha_{A,1}\dot{F}_{A}\left( t \right)$$

$+ higher order terms=0$ (S.3)

whose discrete time analogue is the autoregressive moving-average (ARMA) model

$\Sigma_{j}\varrho_{I,j}T_{I}\left( t-j \right)+\Sigma_{j}\varrho_{O,j}T_{O}\left( t-j \right)+\Sigma_{j}\varrho_{S,j}F_{S}\left( t-j \right)+\Sigma_{j}\varrho_{N,j}F_{N}\left( t-j \right)+\Sigma_{j}\varrho_{A,j}F_{A}\left( t-j \right)=0.$ (S.4)

In the simplest case one can omit constraints on the supply of space conditioning services and solve eq. (S.3) for the profile of $F_{A}$ that maintains indoor temperature at a set point, $T^{\dagger}$ ($T_{I}\left( t \right)=T^{\dagger}\Rightarrow\dot{T}_{I}\left( t \right)=0$). Note that when $T_{O}\left( t \right)=T_{I}\left( t \right)=T^{\dagger}$, $F_{A}\left( t \right)=0$ so that cooling energy use is minimized. More realistic endogenous air conditioner cycling can be introduced through, for example, engineering relationships that approximate the supplied cooling load as a function of the set point temperature, indoor temperature, occupancy and HVAC system capacity ($K_{A}$): $F_{A}\left( t \right)=\phi[T^{\dagger}$,$T_{I}\left( t \right),F_{N}\left( t \right),K_{A}]$. The corresponding air conditioner electricity demand can then be calculated based on the time path of endogenous indoor temperatures, which is the advantage of computational building energy simulations (see, e.g., the review by Ali et al, 2021). Regardless, (S.4) drives home that $F_{A}$ responds to solar radiation, building occupancy and outdoor temperature instantaneously and with one or more lags. The larger the magnitudes of the coefficients $\varrho_{O,j}$, $\varrho_{N,j}$, $\varrho_{S,j}$ and particularly $\varrho_{I,j}$ for $j>0$, the stronger the effect of forcings at previous time steps on contemporaneous indoor temperature, cooling demand and associated electricity use.

For each of our cities, aggregate electric load is the superposition of the solution to (S.4) over thousands to millions of heterogeneous structures, each with unobserved and likely distinct transfer coefficients, occupancy patterns, HVAC capacities, and indoor temperatures based on occupants’ thermal comfort preferences, but facing common time profiles of ambient temperature and solar forcing. Our statistical model captures the average covariation between the unobserved component of electricity consumption that is attributable to $F_{A}$ summed over all buildings, and current and lagged values of $T_{O}$ and $F_{S}$, over hours of the year. Wood et al (2015) illustrate the empirical implementation of eq. (S.4). Our empirical specification omits the autoregressive component of their model, principally because dynamic load projections based on the resulting fitted model are sensitive to initial hourly loads—whose values decades in the future are a key imponderable.

*Generalized additive models*

Demand response functions of log per capita hourly electric load to hourly and lagged temperatures in each city are estimated using a generalized additive model (GAM) implemented via the R package ‘mgcv’ (Wood, 2017). The GAM is estimated via penalized maximum likelihood that allows us to flexibly incorporate nonlinear transformations of predictor variables using smooth functions. Formally, the model can be represented as

$g\left( E\left( y \right) \right)=f_{1}\left( x_{1} \right)+\ldots+f_{p}\left( x_{p} \right)$, (S.5)

where $y$ is a response variable with values drawn from some exponential family distribution (in this case, Gaussian), $g(\cdot)$, $x_{1},\ldots,x_{p}$ denote $p$ predictor variables, and $f(\cdot)$ is a smooth function of covariates, represented as the sum of basis functions:

$f_{p}\left( x_{p} \right)= \Sigma_{j=1}^{k(p)}b_{j}\left( x_{p} \right)\theta_{j,p}$. (S.6)

Here, $b_{j}(\cdot)$ is the $j^{th}$ basis function, or spline, with coefficient, or weight, $\theta_{j,p}$, to be estimated, and $k$ indexes the number of knots, which determines the function’s maximum complexity, or degree of smoothing. The degree of smoothing can be regulated by altering the dimension of the basis (e.g., specifying $b$ as a polynomial, cubic spline, B-spline, etc.) or by applying a ‘wiggliness’ penalty to the basis function weights (a penalized regression spline):

$J_{p}=\int_{0}^{1} \left[ f_{p}^{''}\left( x_{p} \right) \right]^{2}dx_{p}=\boldsymbol{\theta}_{p}^{'}\boldsymbol{S}_{p}\boldsymbol{\theta}_{p}$, (S.7)

where $\boldsymbol{S}$ is a penalty matrix of known coefficients. Together, eqs. (S.5) and (S.6) imply an inherently linear model, $\boldsymbol{y}=\boldsymbol{X\theta}$, in which the row of the model matrix corresponding to the $i$^th^ observation is

$$\boldsymbol{X}_{i}=\left[ b_{1}\left( x_{1i} \right),b_{2}\left( x_{1i} \right),\ldots,b_{k\left( 1 \right)}\left( x_{i} \right),b_{1}\left( x_{2i} \right),\ldots,b_{k(p)}\left( x_{pi} \right) \right]$$

The tradeoff between model fit and model smoothness is controlled by a smoothing parameter, $\lambda$, whereby $\lambda\to\infty$ results in a (over-smoothed) linear model while $\lambda=0$ results in an (under-smoothed) unpenalized regression spline estimate. The penalized regression spline fitting problem thus minimizes:

$\left| \boldsymbol{y}-\boldsymbol{X\theta} \right|^{2}+\Sigma_{m=1}^{p} \lambda_{m}J_{m}$ (S.8)

Our empirical model specifies $b$ as thin-plate splines which assume that the degree of smoothing in any covariate is isotropic. The optimal value of $\lambda$ is found by solving (S.8) iteratively in order to minimize a generalized cross-validation (GCV) score. For details, see Wood (2017: 128-131, 150-156, 168-184).

We model the interaction between contemporaneous and lagged values of meteorological predictors (temperature, dew point temperature and downward shortwave radiation) over a 6-hour window through the use of tensor product smooths (Wood 2017: 158-163). The tensor product smooth facilitates the interaction of its constituent covariates (e.g., contemporaneous hourly temperature, temperature one hour prior, temperature two hours prior, etc.) through the use of ‘marginal’ bases that each have their own ‘marginal’ smoothness penalty to produce a single model matrix for use as the smooth function, where the basis dimension is the product of the basis dimensions of the marginal smooths (Wood, 2006; Pederson et al, 2019).

*Data quality*

Our electricity load series come from Cohen et al (2015), who collect hourly load data for 36 cities in North America, Africa, and Asia for various years over the period 2003-2018. Load profiles from earlier years are unlikely to reflect the most current state of energy demand in some of these urban areas as energy systems evolve with increasing rates of electrification in developing country cities and energy efficiency improvements in developed country cities. In addition, for many tropical, developing country cities in the sample, the time periods on which data are available are limited (ranging from <1-2 years) and dated (up to 10 years) which may influence the shape of the demand response surfaces described in our results. To assess the robustness of the temperature-load response relationship, we select three tropical cities (Abidjan, Accra, and Mindelo) and compare each demand response function after 1) removing outliers based on the Cook’s D statistic, 2) excluding hourly electric load observations below the 0.05^th^ and above the 99.5^th^ percentile and 3) excluding these same percentiles of observations for hourly temperatures. The resulting splines (Figure S14) are qualitatively similar, with relatively inelastic non-monotonic increases across filtering techniques, demonstrating that the temperature-response relationship is robust despite the described confines of some of these data. In addition, where possible we made substitutions to the data sources used. Specifically, additional and/or more recent years of data were replaced for both Singapore and Tokyo (years and source, Table S7). A comparison of the splines in each city before and after this substitution (Figure S15) reiterate that despite potential limitations in data quality, the shape of demand in response to temperature remains stable.

**Figure S14**

Robustness testing of demand response functions in Abidjan, Accra, and Mindelo. From left to right: ‘original’ splines in main text, splines after filtering for outliers, splines after filtering electric load observations >0.05^th^ percentile and >99.5^th^ percentile, splines after filtering temperature observations >0.05^th^ percentile and >99.5^th^ percentile


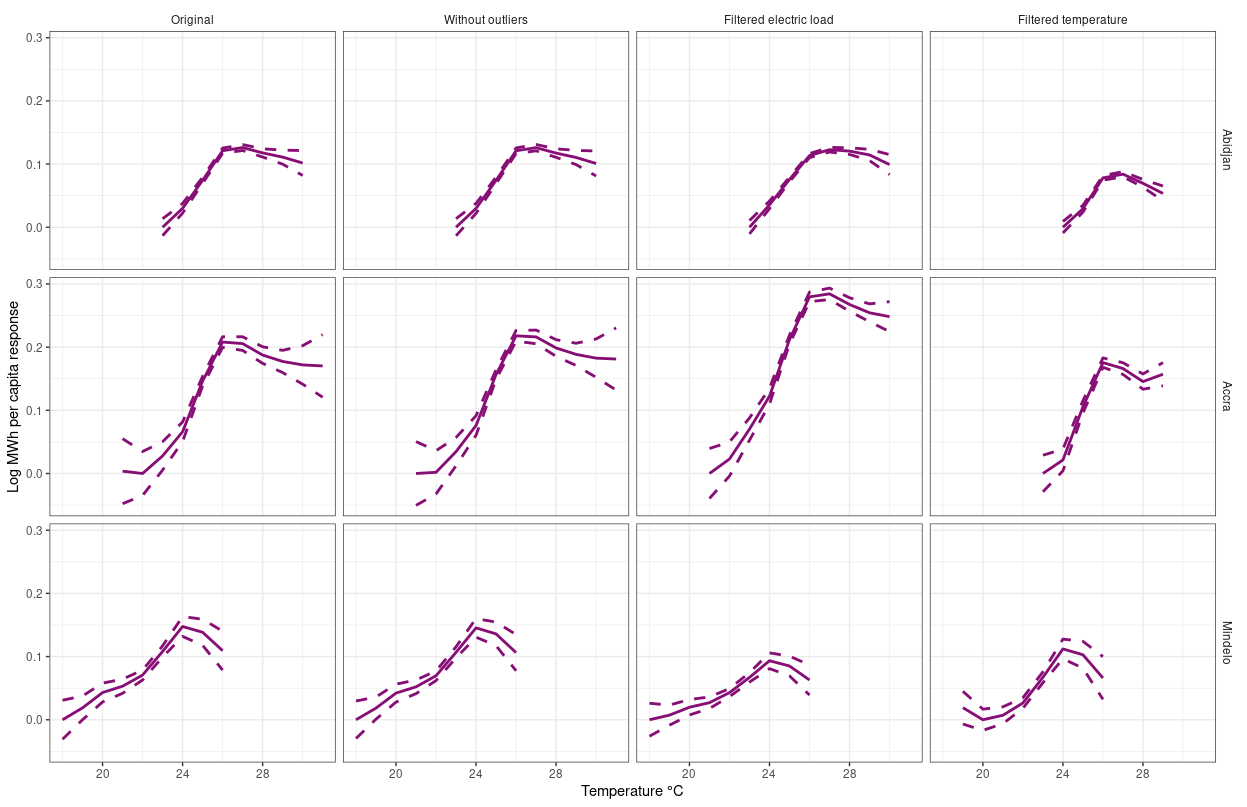


**Figure S15**

Comparison of demand response functions in Singapore and Tokyo without (**a**) and with (**b**) substitution of additional and/or more recent hourly load data.


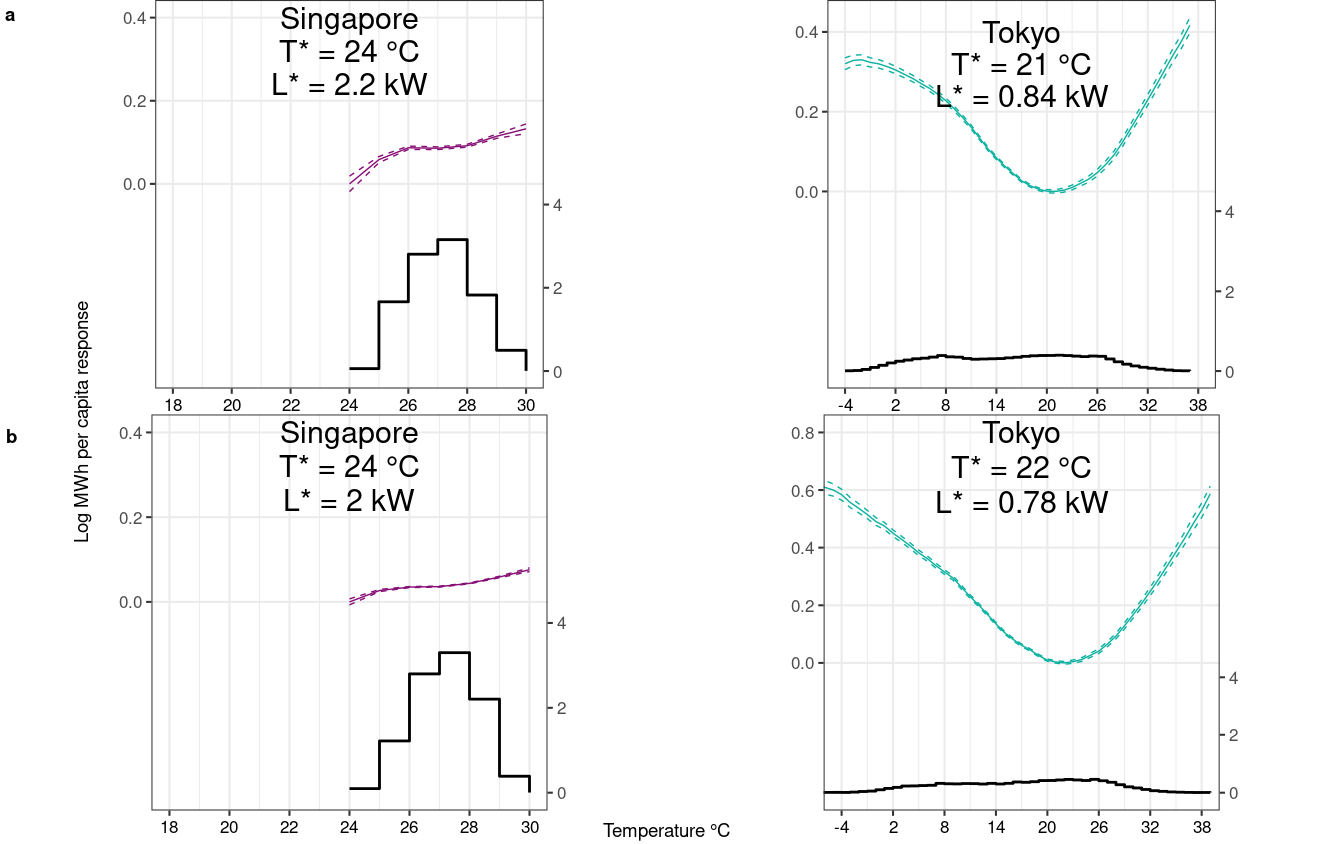


*Sensitivity analysis*

To assess the sensitivity of our empirical model, we evaluate the percentage by which projections of per-capita hourly demand for electricity shifts relative to its conditional mean due to variation (plus or minus one standard deviation) in mean seasonal temperature, dew point temperature (reflecting humidity), solar radiation, and wind speed over warm and cool seasons (in the northern hemisphere, June-July-August, JJA, and December-January-February, DJF, respectively). Variation in mean seasonal temperature induces decreases in cool season electricity demand alongside much larger increases during the warm season across most temperate cities, a pattern that is largely mirrored for dew point temperature. The tropics, owing to their generally warm climates, tend to respond similarly for both variables, albeit at lesser magnitudes and small increases (rather than reductions) during the cool season alongside greater increases during the warm season. Variation in solar radiation received at the surface drives demand upwards for the majority of cities, with small to moderate reductions in Abidjan, Accra, Amman, Beirut, Chandigarh, Delhi, Honolulu, Manila, and Singapore—we speculate that this is likely a result of unobserved building heat transfer characteristics that lead to comfortable temperatures requiring less heating during the winter, and unobserved dynamics in building occupancy that result in less electricity use on sunny days during the summer. Variations in wind speed have minimal impacts on demand across temperate and tropical cities alike.

We additionally evaluate the sensitivity of our results to the correlation amongst meteorological variables (specifically, wind speed and solar irradiance). Pearson correlation coefficients are calculated for each city (Table S5). Overall, these variables are only moderately correlated in six cities. To assess the impact of this correlation upon cities’ temperature-demand response function, we estimate our empirical model excluding wind speed, and compare the resulting changes in our main results, i.e., the shift in relative annual total and peak per capita demand. Across GCMs and cities, the median difference in relative demand ranges from -0.16 to 0.3 percentage points for annual total per capita demand and -0.42 to 0.92 percentage points for peak per capita demand (Table S6), demonstrating that our results are not meaningfully impacted by this correlation.

**Table S4** Semi-elasticities of the response of per capita kW demand to variation in mean seasonal (winter – December, January, February; summer – June, July, August) meteorological variables.

**
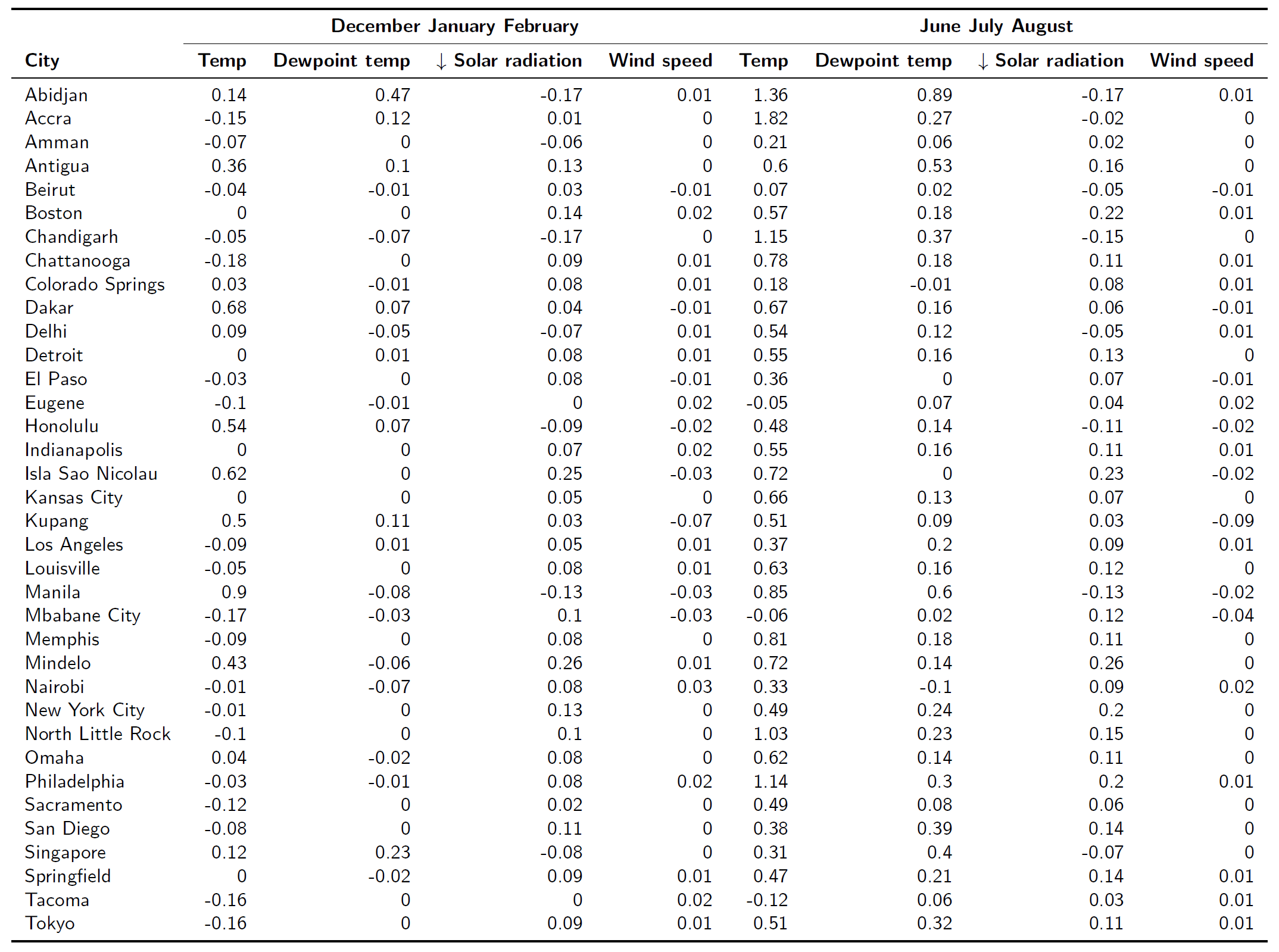
**

**Table S5**

Correlation of wind speed and solar irradiance (lowest to highest)

| **City** | **Correlation** |
| --- | --- |
| Antigua | -0.13179 |
| Boston | -0.10356 |
| Tacoma | -0.06729 |
| Louisville | -0.04478 |
| Dakar | -0.0307 |
| Mindelo | -0.00927 |
| Honolulu | -0.00589 |
| Isla Sao Nicolau | -0.00092 |
| Memphis | 0.004107 |
| Detroit | 0.004503 |
| North Little Rock | 0.012566 |
| Singapore | 0.012872 |
| Indianapolis | 0.016248 |
| Chattanooga | 0.035023 |
| Philadelphia | 0.05134 |
| Springfield | 0.054594 |
| New York City | 0.0592 |
| Abidjan | 0.061248 |
| Kansas City | 0.073927 |
| Sacramento | 0.085447 |
| Tokyo | 0.109216 |
| Omaha | 0.118028 |
| Eugene | 0.134977 |
| Kupang | 0.171439 |
| Colorado Springs | 0.215575 |
| Accra | 0.215608 |
| Beirut | 0.232129 |
| Delhi | 0.233139 |
| El Paso | 0.234151 |
| Mbabane City | 0.275108 |
| Manila | 0.318541 |
| Nairobi | 0.364331 |
| Chandigarh | 0.373109 |
| San Diego | 0.395266 |
| Amman | 0.395519 |
| Los Angeles | 0.528092 |

**Table S6**

Median difference (percentage points) across GCMs in the percentage change of annual total and peak per capita demand when excluding wind speed from the empirical model.

| **City** | **Total** | **Peak** |
| --- | --- | --- |
| Abidjan | -0.04 | 0.06 |
| Accra | 0.17 | -0.04 |
| Amman | -0.01 | -0.06 |
| Antigua | 0.02 | -0.06 |
| Beirut | -0.03 | -0.28 |
| Boston | 0.01 | -0.43 |
| Chandigarh | 0.00 | -0.01 |
| Chattanooga | -0.05 | -0.06 |
| Colorado Springs | -0.02 | 0.16 |
| Dakar | -0.11 | -0.04 |
| Delhi | -0.04 | -0.03 |
| Detroit | 0.07 | -0.09 |
| El Paso | 0.12 | 0.20 |
| Eugene | 0.03 | -0.92 |
| Honolulu | 0.10 | -0.16 |
| Indianapolis | -0.07 | 0.17 |
| Isla Sao Nicolau | -0.25 | 0.02 |
| Kansas City | -0.01 | -0.02 |
| Kupang | 0.08 | 0.42 |
| Los Angeles | 0.06 | 0.42 |
| Louisville | -0.07 | -0.24 |
| Manila | -0.30 | 0.16 |
| Mbabane City | 0.03 | 0.10 |
| Memphis | 0.04 | 0.03 |
| Mindelo | 0.10 | 0.04 |
| Nairobi | -0.01 | -0.08 |
| New York City | -0.01 | -0.55 |
| North Little Rock | -0.01 | -0.01 |
| Omaha | 0.12 | -0.09 |
| Philadelphia | -0.14 | -0.69 |
| Sacramento | 0.00 | 0.00 |
| San Diego | 0.00 | -0.01 |
| Singapore | -0.03 | 0.20 |
| Springfield | 0.03 | -0.54 |
| Tacoma | -0.07 | -0.53 |
| Tokyo | -0.18 | -0.16 |

**Table S7**

Electricity Load Data

| **City** | **Years** | **Source** |
| --- | --- | --- |
| Abidjan | 2010-2013 | Cohen et al, 2015^[[2]](#endnote-1)^ |
| Accra | 2013-2014 | Cohen et al, 2015 |
| Amman | 2011-2014 | Cohen et al, 2015 |
| Antigua | 2011 | Cohen et al, 2015 |
| Beirut | 2011-2014 | Cohen et al, 2015 |
| Boston | 2003-2018 | ISO New England Inc.^[[3]](#endnote-2)^ |
| Chandigarh | 2011-2013 | Cohen et al, 2015 |
| Chattanooga | 2006-2013 | Cohen et al, 2015 |
| Colorado Springs | 2006-2013 | Cohen et al, 2015 |
| Dakar | 2011-2014 | Cohen et al, 2015 |
| Delhi | 2011-2013 | Cohen et al, 2015 |
| Detroit | 2006-2008 | Cohen et al, 2015 |
| El Paso | 2006-2013 | Cohen et al, 2015 |
| Eugene | 2006-2013 | Cohen et al, 2015 |
| Honolulu | 2006-2013 | Cohen et al, 2015 |
| Indianapolis | 2006-2008 | Cohen et al, 2015 |
| Isla Sao Nicolau | 2013 | Cohen et al, 2015 |
| Kansas City | 2006-2013 | Cohen et al, 2015 |
| Kupang | 2014 | Cohen et al, 2015 |
| Los Angeles | 2006-2013 | Cohen et al, 2015 |
| Louisville | 2006-2013 | Cohen et al, 2015 |
| Manila | 2011-2013 | Cohen et al, 2015 |
| Mbabane City | 2012-2014 | Cohen et al, 2015 |
| Memphis | 2006-2013 | Cohen et al, 2015 |
| Mindelo | 2013 | Cohen et al, 2015 |
| Nairobi | 2011-2013 | Cohen et al, 2015 |
| New York City | 2006-2012 | Cohen et al, 2015 |
| North Little Rock | 2010-2013 | Cohen et al, 2015 |
| Omaha | 2006-2013 | Cohen et al, 2015 |
| Philadelphia | 2009-2011 | Cohen et al, 2015 |
| Sacramento | 2006-2013 | Cohen et al, 2015 |
| San Diego | 2006-2013 | Cohen et al, 2015 |
| Singapore | 2012-2018 | Energy Market Authority of Singapore^[[4]](#endnote-3)^ |
| Springfield, IL | 2011-2013 | Cohen et a., 2015 |
| Tacoma | 2006-2013 | Cohen et al, 2015 |
| Tokyo | 2016-2018 | TEPCO Tokyo Electric Power Company Holdings^[[5]](#endnote-4)^ |

**Table S8**

Population Data

| **City** | **Load (MW)** |
| --- | --- |
| Abidjan | UN DESA 2018 World Urbanization Prospects^[[6]](#endnote-5)^ |
| Accra | UN DESA 2018 World Urbanization Prospects |
| Amman | UN DESA 2018 World Urbanization Prospects |
| Antigua | Antigua and Barbuda 2011 Population and Housing Census^[[7]](#endnote-6)^ |
| Beirut | UN DESA 2018 World Urbanization Prospects |
| Boston | US BEA 2020^[[8]](#endnote-7)^ |
| Chandigarh | UN DESA 2018 World Urbanization Prospects |
| Chattanooga | US BEA 2020 |
| Colorado Springs | US BEA 2020 |
| Dakar | UN DESA 2018 World Urbanization Prospects |
| Delhi | UN DESA 2018 World Urbanization Prospects |
| Detroit | US BEA 2020 |
| El Paso | US BEA 2020 |
| Eugene | US BEA 2020 |
| Honolulu | US BEA 2020 |
| Indianapolis | US BEA 2020 |
| Isla Sao Nicolau | Cabo Verde Statistical Yearbook 2015^[[9]](#endnote-8)^ |
| Kansas City | US BEA 2020 |
| Kupang | Urban Climate Risk Management Plan - City of Kupang 2015^[[10]](#endnote-9)^ |
| Los Angeles | US BEA 2020 |
| Louisville | US BEA 2020 |
| Manila | UN DESA 2018 World Urbanization Prospects |
| Mbabane City | UN Country Profile - Swaziland^[[11]](#endnote-10)^ ^[[12]](#endnote-11)^ |
| Memphis | US BEA 2020 |
| Mindelo | Economist Intelligence Unit – Cabo Verde Summary^[[13]](#endnote-12)^ |
| Nairobi | UN DESA 2018 World Urbanization Prospects |
| New York City | US BEA 2020 |
| North Little Rock | US BEA 2020 |
| Omaha | US BEA 2020 |
| Philadelphia | US BEA 2020 |
| Sacramento | US BEA 2020 |
| San Diego | US BEA 2020 |
| Singapore | UN DESA 2018 World Urbanization Prospects |
| Springfield, IL | US BEA 2020 |
| Tacoma | US BEA 2020 |
| Tokyo | UN DESA 2018 World Urbanization Prospects |

**References**

Ali, U., M. Shamsi, C. Hoare, E. Mangina and J. O’Donnell (2021). Review of urban building energy modeling (UBEM) approaches, methods and tools using qualitative and quantitative analysis, *Energy and Buildings* **246**: 111073.

Auffhammer, M. and Aroonruengsawat, A. Simulating the impacts of climate change, prices and population on California’svresidential electricity consumption*. Climatic Chang*e **109**, 191–210, DOI: 10.1007/s10584-011-0299-y (2011).

Hess, D.J., and Gentry, H. 100% renewable energy policies in US cities: strategies, recommendations, and implementation challenges. *Sustainability: Science, Practice and Policy* **15,** 1, DOI:10.1080/15487733.2019.1665841 (2019).

Jayathissa, P., M. Luzzatto, J. Schmidli, J.Hofer, Z. Nagy and A.Schlueter (2017). Optimising building net energy demand with dynamic BIPV shading, *Applied Energy* **202**: 726-735.

Murdock, H. E., et al. Renewables 2019 global status report. ISBN 978-3-9818911-7-1 (REN21 Secretariat, 2019).

Pedersen, E.J., Miller, D.L., Simpson, G.L. and Ross, N. Hierarchical generalized additive models in ecology: an introduction with mgcv. *PeerJ,* **7**, p.e6876, DOI: 10.7717/peerj.6876 (2019).

Rabl, A. (1988). Parameter Estimation in Buildings: Methods for Dynamic Analysis of Measured Energy Use, *Journal of Solar Energy Engineering* **110**: 52-66.

Steinberg, D. C., et al. Decomposing supply-side and demand-side impacts of climate change on the US electricity system through 2050. *Climatic Change* **158**, 2, DOI: 10.1007/s10584-019-02506-6 (2020).

van Ruijven, B. J., De Cian, E. and Sue Wing, I. Amplification of future energy demand growth due to climate change. *Nat. Comm*. **10**, 2762, DOI: 10.1038/s41467-019-10399-3 (2019).

Wenz, L., Levermann, A. and Auffhammer, M. North–south polarization of European electricity consumption under future warming. *PNAS,* DOI: 10.1073/pnas.1704339114 (2017).

Wood, S.N. Low rank scale invariant tensor product smooths for generalized additive mixed models. *Biometrics* **62**, 4,1025-1036, DOI: 10.1111/j.1541-0420.2006.00574.x (2006).

Wood S.N. Generalized Additive Models: An Introduction with R (2nd edition). (Chapman and Hall/CRC Press, 2017).

Wood, S.N., Y. Goude and S. Shaw (2015). Generalized additive models for large data sets, Applied Statistics Series C 64 (Part1): 139-155.

1. See, e.g., <https://www.forbes.com/sites/energyinnovation/2019/07/22/as-cities-begin-banning-natural-gas-states-must-embrace-building-electrification-with-smart-policy/>. [↑](#footnote-ref-1)
2. Cohen, E., Modi V., Torbey H., Piccirelli M., and Tian Y. Global Trends in Urban Energy Use. Working Paper of the Sustainable Engineering Lab, Columbia University, Available online: <http://ecohen4.github.io/Energy/Global_Trends_v4.html> (2015). [↑](#endnote-ref-1)
3. ISO New England, Inc. Energy, Load, and Demand Reports: SMD Hourly Data <https://www.iso-ne.com/isoexpress/web/reports/load-and-demand/-/tree/zone-info>. [↑](#endnote-ref-2)
4. Singapore Energy Market Authority (EMA). Statistics: Half-hourly System Demand Data <https://www.ema.gov.sg/statistic.aspx?sta_sid=20140826Y84sgBebjwKV>.Tokyo Electric Power Company Holdings (TEPCO). Download past electricity demand data <https://www.tepco.co.jp/en/forecast/html/download-e.html>. [↑](#endnote-ref-3)
5. Tokyo Electric Power Company Holdings (TEPCO). Download past electricity demand data <https://www.tepco.co.jp/en/forecast/html/download-e.html>. [↑](#endnote-ref-4)
6. United Nations, Department of Economic and Social Affairs, Population Division. World Urbanization Prospects: The 2018 Revision <https://population.un.org/wup/DataQuery/> (2019). [↑](#endnote-ref-5)
7. Government of Antigua and Barbuda. Antigua and Barbuda 2011 Population and Housing Census: a Demographic Profile <https://statistics.gov.ag/wp-content/uploads/2017/11/2011-Antigua-and-Barbuda-Population-and-Housing-Census-A-Demographic-Profile.pdf> (2017). [↑](#endnote-ref-6)
8. United States Bureau of Economic Analysis (US BEA). Personal Income, Population, Per Capita Personal Income (CAINC1) <https://apps.bea.gov/> (2020). [↑](#endnote-ref-7)
9. Instituto Nacional de Estatística Cabo Verde. Statistical Yearbook 2015 <http://ine.cv/wp-content/uploads/2017/02/statistical-yearbook-cv-2015_en.pdf> (2015). [↑](#endnote-ref-8)
10. United Nations Development Programme (UNDP). Kupang, Indonesia: Urban Climate Risk Management Plan (UCRMP) <http://www.kotakita.org/publications-docs/UCRMP_Kupang_ENG.pdf> (2015). [↑](#endnote-ref-9)
11. United Nations. Country Profile – Swaziland [http://data.un.org/CountryProfile.aspx/](http://data.un.org/CountryProfile.aspx/_Images/CountryProfile.aspx?crName=Swaziland) (2020). [↑](#endnote-ref-10)
12. Mbabane City population calculated from UN data using 2014 population and UN data 1.3% annual urban growth rate [↑](#endnote-ref-11)
13. The Economist Intelligence Unit (EIU). Cabo Verde. <https://country.eiu.com/cabo-verde> (2018). [↑](#endnote-ref-12)
